# Toward Solar-Powered Growth of Autotrophic *Escherichia coli* Using Photoelectrochemistry

**DOI:** 10.1021/jacs.6c03677

**Published:** 2026-05-19

**Authors:** Lin Su, Celine Wing See Yeung, Eliya Milshtein, Beverly Qian Ling Low, Yongpeng Liu, Ron Milo, Erwin Reisner

**Affiliations:** † Yusuf Hamied Department of Chemistry, 2152 University of Cambridge, Lensfield Road, Cambridge CB2 1EW, U.K.; ∥ Department of Biochemistry, School of Biological and Behavioural Sciences, 4617 Queen Mary University of London, Mile End Road, London E1 4NS, U.K.; § Department of Plant and Environmental Sciences, 34976 Weizmann Institute of Science, Rehovot 7610001, Israel

## Abstract

Integrated coupling of renewable energy sources with microbial CO_2_ fixation remains a major challenge in carbon-neutral biomanufacturing. Here, a biohybrid design is presented that combines a semiartificial leaf for solar-powered conversion of CO_2_ into formate with an autotrophic *Escherichia coli* (*E. coli*) that is engineered to use formate as an energy source to produce biomass. First, adaptive laboratory evolution was employed to enhance formate consumption and overcome slow autotrophic growth. Second, electrode-microbe compatibility was established, showing that the adapted strain can grow directly using formate electrochemically generated from CO_2_ by an enzyme-modified cathode. Third, the electrical energy source was replaced with simulated sunlight, developing a biophotoelectrochemical device to support *E. coli* growth. Finally, an integrated platform consisting of a semiartificial leaf and autotrophic *E. coli* was designed, which couples solar CO_2_-to-biomass conversion and O_2_ evolution, replicating natural photosynthesis.

## Graphical abstract

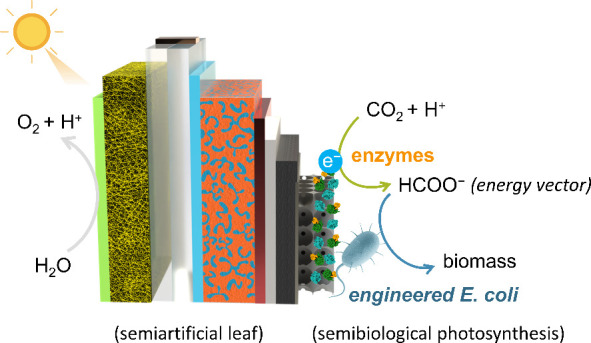

The solar-driven reduction of CO_2_ into single-carbon compounds such as formate provides an efficient and sustainable entry point for green chemical production. The simple platform chemical formate can then be upgraded into complex multicarbon products through formate-utilizing bacteria that give access to an emerging approach known as the ″formate bioeconomy″, which benefits from the integration of abiotic with biological processes.^1–4^ Natural autotrophs such as *Cupriavidus necator* ^5,6^ and *Methylobacterium extorquens* ^7^ feed on formate, using it as a source of reducing power to fix CO_2_ via the Calvin or serine cycle. However, these organisms are difficult to engineer and have a narrow product range.

To overcome these limitations, *Escherichia coli* (*E. coli*) was selected as the prototype chassis, with a well-characterized physiology and genetic toolbox. Its amenability to adaptive laboratory evolution (ALE) and established use as an industrial production host make it a suitable chassis for rewiring metabolism toward formate-driven CO_2_ assimilation.^8^ A series of studies have introduced modules such as the reductive glycine pathway (rGlyP), C1-metabolism and serine biosynthesis, as well as non-native energy (formate dehydrogenase, FDH) and carbon-fixing (Calvin-Benson Bassham cycle, CBB) functions into *E. coli*, supported by ALE.^9–14^ These efforts ultimately led to autotrophic *E. coli*, where all biomass carbon comes from CO_2_ with the energy provided by formate,^10,11^ and a formatotrophic *E. coli* where all carbon and energy come from formate.^12^

The next key step in the development of sustainable biorefineries is to connect this metabolic capability to renewably synthesized formate.^1,15–17^ Early attempts to combine electro- or photocatalytic formate generation with microbial systems highlighted this promise,^9,10,18–22^ but these systems typically lacked stable catalyst–microbe interfaces,^23,24^ suffered from poor electron-to-carbon efficiency, released toxic metal ions that damaged bacterial cells, or failed to sustain continuous growth without adding stoichiometric organic supplements as an electron, energy or carbon source (Table S1).^9–14,25–32^ Parallel to these formate-centric efforts, other pioneering biohybrid works have relied on intermediate H_2_, CO, or syngas to feed gas-fixing microorganisms, or liquid intermediates such as the carbon source acetate with an external energy supply such as glucose to sustain the growth of engineered bacteria in a separate reactor.^33–37^

Here, these challenges are addressed by linking engineered formate-“eating” *E. coli* with electricity- and light-driven formate synthesis to create an integrated semibiological photosynthesis platform (Figure 1).

**Figure 1:**
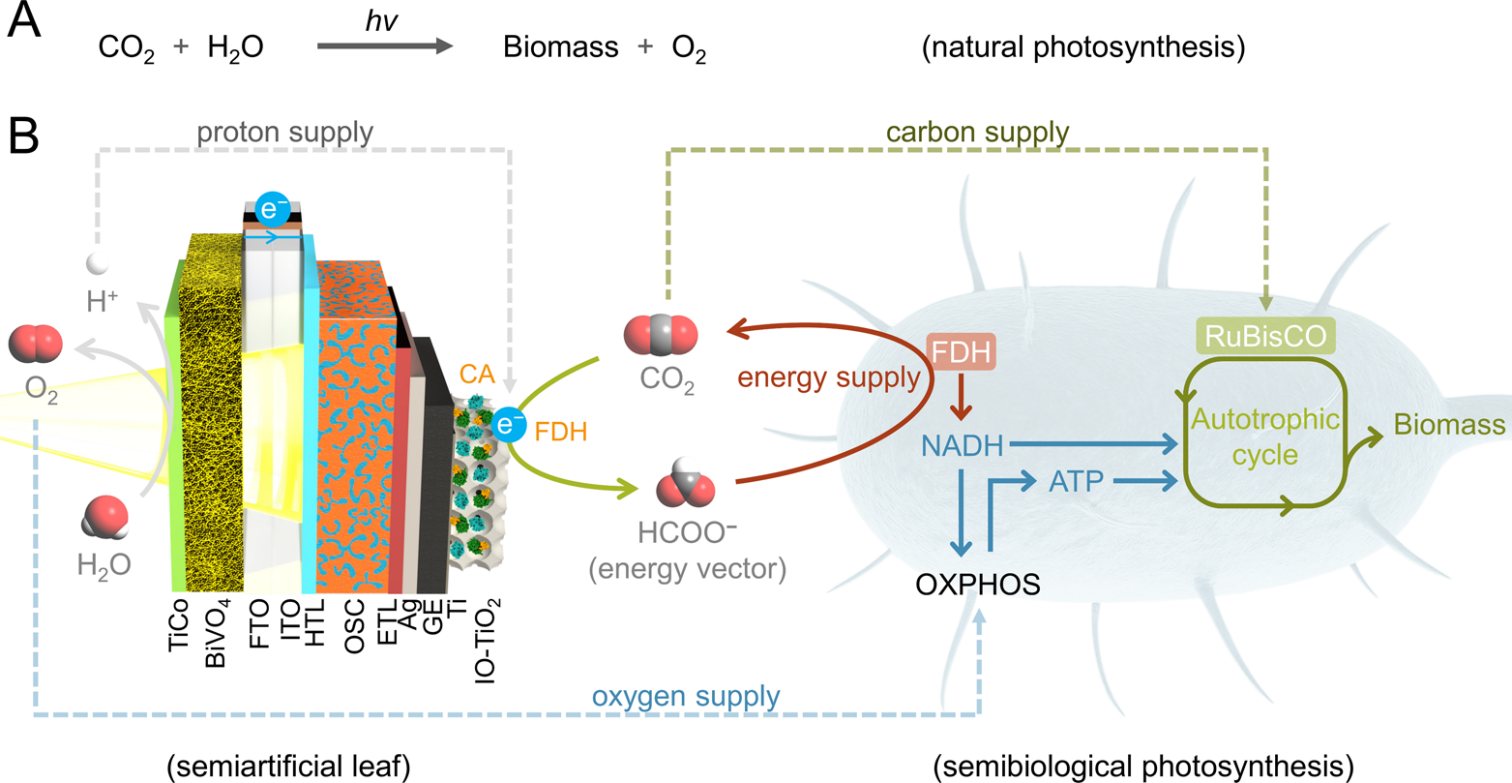
Natural and engineered photosynthesis. (A) Natural photosynthesis: CO_2_ + H_2_O → biomass + O_2_. (B) Semibiological platform (not to scale): a BiVO_4_|TiCo photoanode releases O_2_ for bacterial respiration and is coupled to an OPV|IO-TiO_2_|FDH+CA photocathode for bias-free formate production. Formate fuels engineered *E. coli*, closing the CO_2_ loop. HTL, hole transport layer; OSC, organic semiconductor; ETL, electron transport layer; GE, graphite epoxy encapsulant.

The growth characteristics of a previously engineered autotrophic *E. coli* strain^10,11^ were first optimized through ALE. The parental strain was subjected to 27 consecutive serial transfers (2–15 days for each growth period; total 168 days) in sealed serum bottles containing a mineral medium with 60 mM commercially sourced formate as the sole energy source (5% O_2_ and 10% CO_2_ in N_2_). While the parental strain (#1) required approximately 13 days to reach a stationary phase optical density (OD_600_) of ∼ 0.2 (Figure S1A) following a 1:10 dilution, the evolved strain isolated from the final transfer (#27) achieved a comparable density in just 2 days (Figure 2).

**Figure 2:**
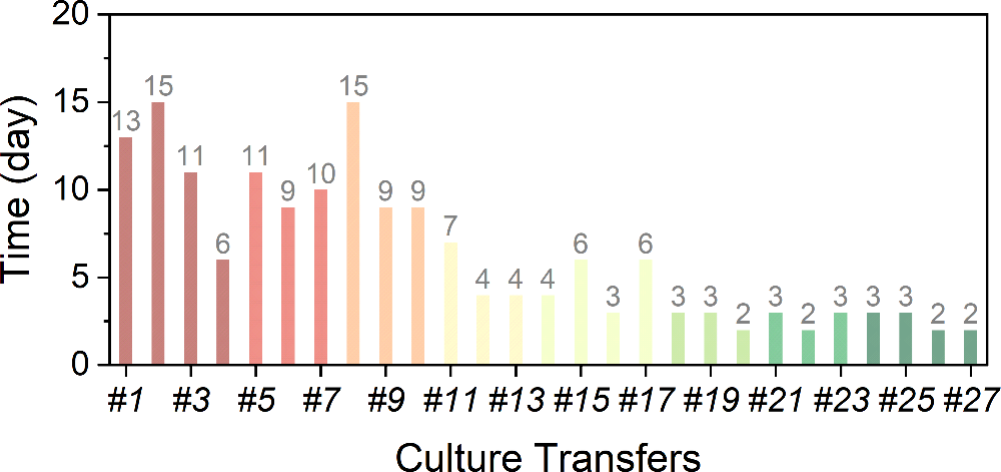
Accelerated autotrophic growth on formate. ALE kinetics over 27 serial transfers; time to stationary phase decreased from ∼13 (#1) to 2 days (#27).

Side-by-side characterization of the parental (#1) and evolved (#27) strains was performed under identical preculturing (first 48 h, 60 mM formate) showed that the evolved strain consumed 21 ± 8 mM formate and reached an OD_600_ of 0.23 ± 0.08, whereas the parental strain remained in a lag phase during this period, exhibiting negligible growth (OD_600_ 0.02) and minimal formate consumption (6 ± 2 mM) (Figure S1B). Both strains converged to comparable final densities (#1: 0.34 ± 0.02; #27: 0.46 ± 0.13), indicating that the evolutionary pressure selected for an earlier onset of metabolic flux rather than enhanced carbon conversion efficiency.

Whole-genome sequencing of the evolved strain (#27) identified a single additional fixed mutation relative to the parental strain (#1): a +T insertion at position 1457 (out of 1500) of *pitA*, which encodes the low-affinity inorganic phosphate transporter (Table S2). To test the effect of this mutation, the +T insertion in *pitA* was reverted in the evolved strain background and growth was compared to that of the evolved strain. Growth was monitored in minimal medium supplemented with formate under an elevated CO_2_ atmosphere. The reversion of *pitA* showed reduced fitness relative to the evolved strain, supporting a functional contribution of the *pitA* mutation (Figure S1C). The +T insertion introduces a frameshift, likely causing some loss of function. Medium alkalinization during growth on formate has been widely reported for formatotrophic *E. coli*.^38,39^ PitA mediates proton-coupled metal-phosphate transport and influences intracellular metal homeostasis under stress conditions.^40,41^ It is therefore hypothesized that the inactivation of PitA helps conserve the proton motive force under the alkaline, energy-limited conditions, thereby improving overall formate utilization by ensuring the generated cellular energy is directed toward autotrophic growth rather than compensatory ion pumping.

Next, the ability of the evolved strain to utilize formate generated directly from CO_2_ was studied. To achieve this, a tungsten-dependent FDH ([W]-FDH) from *Nitratidesulfovibrio vulgaris* Hildenborough (*Nv*H), was immobilized onto hierarchically structured inverse opal TiO_2_ (IO-TiO_2_) electrodes. The electrodes were fabricated on Ti foil by a previously reported template-assisted method (Figure 3A),^42,43^ yielding a hierarchically porous film (∼40 μm thick; ≈710 nm macropores supported by ≈21 nm TiO_2_ anatase nanoparticles verified by SEM imaging; Figure S2).^44,45^ This allowed for a high FDH loading (500 pmol) and formate concentrations sufficient for bacterial growth.

**Figure 3:**
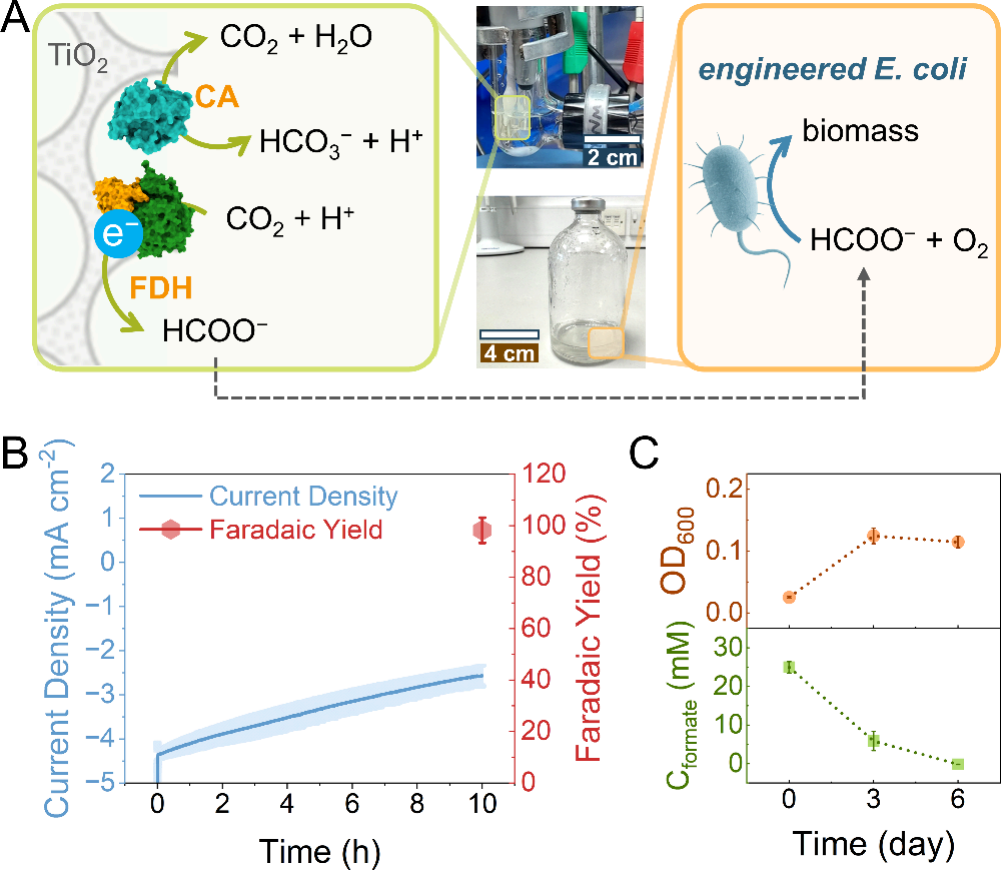
Bioelectrochemical formate synthesis and utilization. (A) IO-TiO_2_|FDH+CA cathode producing formate for biomass growth. (B) CPE (−0.4 V vs RHE) showing current density and formate Faradaic yield. (C) Growth of evolved *E. coli* and formate consumption.

*Nv*H [W]-FDH avoids the use of metal electrodes that may release cytotoxic metal ions, drives selective CO_2_ reduction with minimal overpotential and is resilient to trace amounts of O_2_ (see Supplementary Note 1, Figure S3–S9).^46,47^ Carbonic anhydrase (CA, 100 pmol) was co-immobilized with FDH to accelerate CO_2_ hydration. When FDH reduces CO_2_ to formate, CA buffers the local pH increase by converting CO_2_ and H_2_O into HCO_3_^–^ and H^+^ (Figure 3A), preventing local alkalinization within the porous IO-TiO_2_ electrode, which would result in lower FDH activity and bioelectrochemical performance.^43,48^

Protein film voltammetry of the resulting IO-TiO_2_|FDH+CA cathode in a CO_2_-saturated H-cell containing NaHCO_3_ (50 mM) and KCl (50 mM) at pH 6.45 and room temperature showed an onset potential for CO_2_ reduction at ∼ 0 V vs RHE with *j* = –4.3 mA cm^–2^ at –0.4 V vs RHE (Figure S10–S12).^43^ Controlled potential electrolysis (CPE) at –0.4 V vs RHE over 10 h produced 650 ± 44 μmol_formate_ cm^–2^ with a Faradaic yield of 98 ± 1% and a turnover frequency (TOF) of 6.9 ± 0.5 h^–1^ (Figure 3B, S13).^49^

Dose–response assessments confirmed that formate concentrations below the established 60 mM optimum remain viable for cultivation (Figure S14). The catholyte solution (5 mL) contained 25 ± 1 mM of formate after CPE (pH 7.4 ± 0.1), sufficient to support *E. coli* growth (evolved strain #27) in a separate glass culture reactor supplemented with trace elements under 5% O_2_, 10% CO_2_ in N_2_ at 37 °C (Figure S15, S16).^11^ The culture grew steadily, with the OD_600_ increasing from 0.03 ± < 0.01 to 0.12 ± 0.01 over 3 days (Figure 3C). This growth was directly coupled with the consumption of the electrochemically produced formate, whose concentration decreased from 25 ± 1 mM to 6 ± 2 mM in the same period. The culture reached a stationary phase (OD_600_ of 0.11 ± 0.01) by day 6 when formate was depleted (<1 mM).

To drive formate synthesis with solar energy, IO-TiO_2_|FDH+CA electrodes were interfaced with a conventional-structure organic photovoltaic (OPV) device (Figure 1, 4A).^43^ The all-organic PCE10:EH-IDTBR bulk heterojunction was selected for its 1 V photovoltage^50^ and to avoid metal-ion leaching.^43^ The biophotocathode displayed an onset potential of 1 V vs RHE (Figure S17), with an initial photocurrent density of –3 mA cm^–2^ at 0.6 V vs RHE under AM1.5G irradiation at room temperature (Figure 4B). External quantum efficiency spectra of the OPV photoelectrodes at 0 V and 0.6 vs RHE showed a plateau between 550 and 650 nm, demonstrating a maximum EQE of ∼ 80% consistent with the underlying OPV (Figure S18).

**Figure 4:**
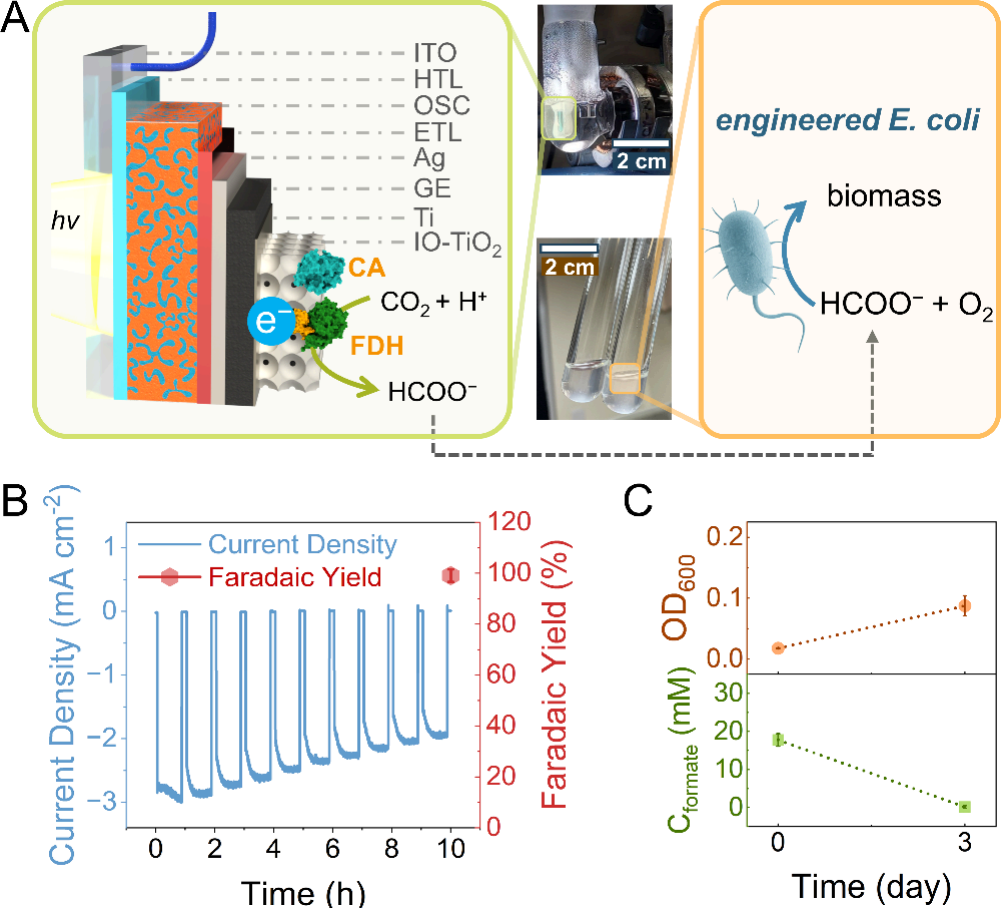
Solar-driven autotrophic growth. (A) OPV-integrated photocathode for formate-mediated biomass growth. (B) Chopped-light photocurrent and formate Faradaic yield at 0.6 V vs RHE. (C) *E. coli* growth and formate consumption in solar reactor effluent.

CPE over 10 h showed good stability with the production of 338 ± 51 μmol_formate_ cm^–2^ (18 ± 2 mM in 5 mL catholyte, pH 7.4 ± 0.2) with a Faradaic yield of 97 ± 4% and a TOF of 4.7 ± 0.7 s^–1^ (Figure S19). The resulting formate concentration was sufficient to support microbial growth at 37 °C. After removing the effluent from the solar reactor, the evolved *E. coli* (#27) was inoculated into this solution and consumed all the photoelectrochemically produced formate, growing to a final OD_600_ of 0.09 ± 0.02 within 3 days (Figure 4C).

To integrate formate synthesis and consumption in a single reactor, a semiartificial leaf device (1.3 cm × 1.3 cm glass slide, photoactive area = 0.25 cm^2^) was constructed for simultaneous CO_2_-to-formate synthesis coupled to O_2_ evolution, integrating *in situ* microbial consumption and biomass growth in a single reactor (Figure 5A). This requires kinetic, chemical, and microenvironmental abiotic–biotic compatibility (Supplementary Note 3). The integration is enabled by a simple NaHCO_3_/KCl electrolyte that, unlike the Good’s buffers and sacrificial reagents common to prior biohybrid systems (Table S1), simultaneously supports BiVO_4_ water oxidation, FDH catalysis, and *E. coli* growth. The solar biohybrid device consisted of an OPV|IO-TiO_2_|FDH+CA photocathode assembled with a BiVO_4_ photoanode (Figure S20),^43^ and was directly immersed into an electrolyte solution containing the evolved *E. coli* strain #27 (starting OD_600_ of 0.01) at 37 °C. In contrast to the decoupled experiments, the solar simulator was equipped with a UV filter (>400 nm) for this one-pot system to avoid possible bacterial damage.

**Figure 5:**
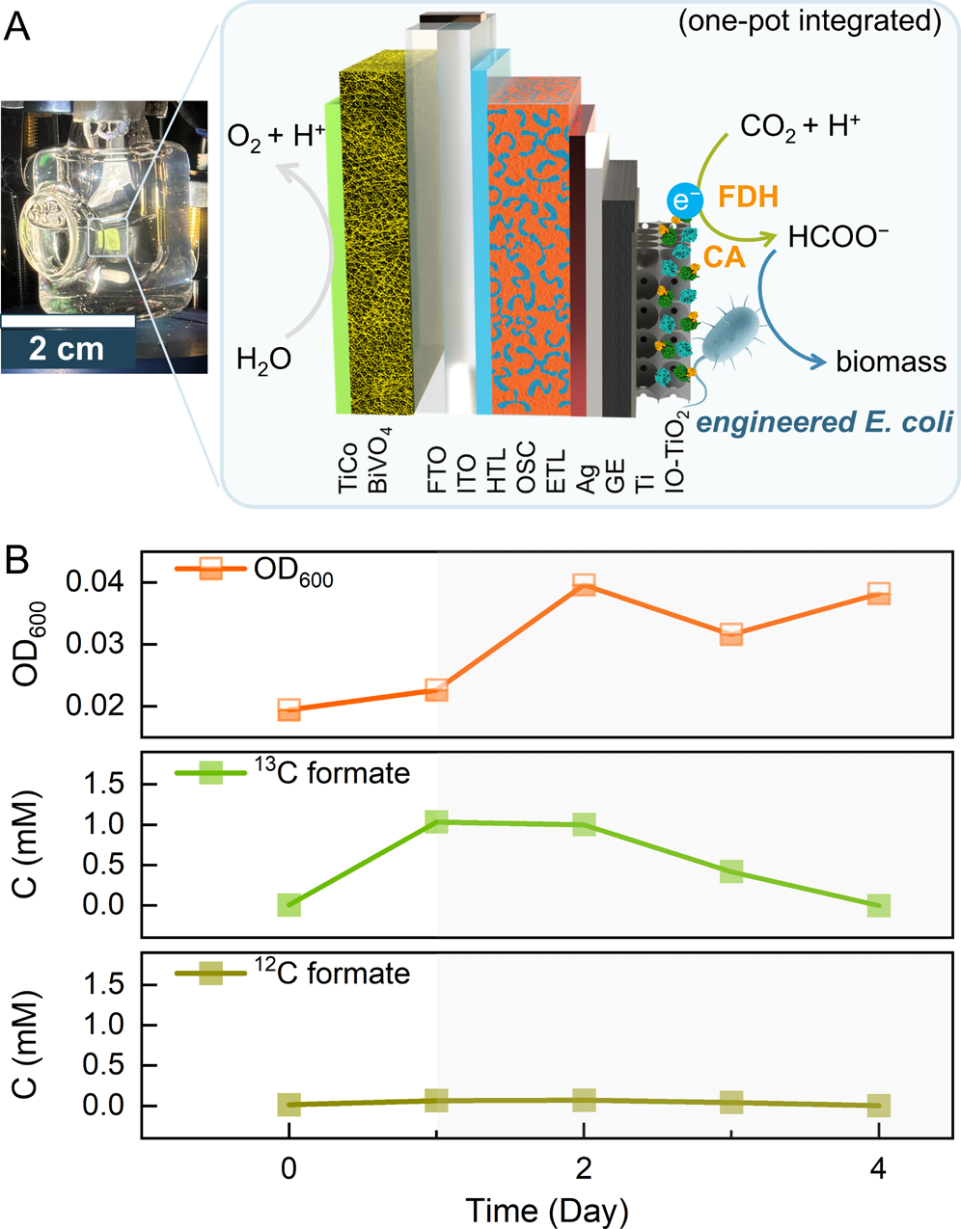
Semibiological photosynthesis drives unassisted autotrophic biomass production. (A) Device configuration pairing an organic photobiocathode and a BiVO_4_ photoanode. (B) *E. coli* growth and ^13^CO_2_ tracing showing light-driven ^13^C-formate fueling biomass production.

Bias-free operation of the semiartificial leaf was conducted over 20 h, yielding 79 ± 34 μmol_formate_ cm^–2^ (4 ± 2 mM in 5 mL) and 26 ± 11 μmol O_2_ cm^–2^ over 20 h (Figure S21–S22). An initial decrease in biomass (Figure S23) was attributed to the absence of trace minerals that could not be added due to FDH inhibition (Figure S15), alongside insufficient O_2_ generation (around 6.5 μmol, Table S3). The O_2_ limitation suppressed aerobic growth (Figure S16), triggering a physiological shift to anaerobic fermentation as evidenced by acetate accumulation (Supplementary Note 2).

To address this challenge, the reactor was further supplemented with trace minerals and provided an optimal O_2_/CO_2_ atmosphere following irradiation, which led to acetate consumption accompanied by a corresponding small increase in bacterial OD_600_ (from 0.02 to 0.04, Figure 5B, S23). Triplicate dark controls showed no formate production and a consistent decline in biomass, confirming that growth in the illuminated reactor is light-dependent (Figure S24). Post-mortem imaging of the electrodes after 3 days revealed no biofilm formation (Figure S25–S26).

To provide further evidence on the carbon pathway from gaseous CO_2_ into biomass, the experiment was replicated using ^13^CO_2_. ^13^C-formate produced was solely derived from the FDH-catalyzed reaction (Figure S27–S29). Because this strain has been shown to synthesize biomass exclusively from CO_2_ using formate as the sole energy source,^10^ the correlation between *in situ* generated ^13^C-formate and bacterial growth (Figure 5B) supports that biomass accumulation was powered by the solar-driven CO_2_ reduction product.

In summary, solar-powered conversion of CO_2_ into biomass was achieved, mirroring the two-stage logic of natural photosynthesis: in the light phase, the semiartificial leaf (abiotic thylakoid) stores solar energy as formate and releases O_2_, analogous to ATP and NADH generation; in the dark, the engineered *E. coli* consumes formate and CO_2_ to build biomass, analogous to carbon fixation in plants (biotic Calvin cycle). This “formate bioeconomy” approach uses engineered *E. coli*, with potential to produce chemical products,^1^ while reducing reliance on sugar feedstocks.^2–4^ Distinct from recent biohybrid systems that utilize biosynthesized acetate as a carbon feedstock,^35,36^ our platform uses CO_2_ as the carbon input alongside the energy carrier formate, offering a sustainable strategy for the synthesis of organic products. Remaining challenges include^51,52^ O_2_ and nutrient management, long-term cyclic stability, and system scalability.

## Supplementary Material



## Data Availability

The data that support the findings of this study can be accessed through the University of Cambridge data repository: 10.17863/CAM.129813

## Notes

Experimental details; supplementary notes on glycerol removal, hypoxia-induced acetate production, and one-pot integration rationale; bacterial cultivation, adaptive laboratory evolution, and whole-genome sequencing; FDH purification; electrode and device fabrication; SEM and EDX images; ^1^H NMR spectra; protein film voltammetry; controlled potential electrolysis profiles; external quantum efficiency spectra; ^13^C isotopic tracing data (Supplementary Notes 1–3; Figures S1–S29 and Tables S1–S4) (PDF)
