# Supplementary material for: Toward Solar-Powered Growth of Autotrophic Escherichia coli Using Photoelectrochemistry
Source: J Am Chem Soc. 2026 May 19;148(21):21198–203. doi: 10.1021/jacs.6c03677 (PMC13244470; doi:10.1021/jacs.6c03677)
Supplement: Supplementary file 1 [file ja6c03677_si_001.pdf]

Supporting Information for

Towards solar-powered growth of autotrophic *Escherichia coli* using photoelectrochemistry

Lin Su[1,2,‡], Celine Wing See Yeung[1,‡], Eliya Milshtein[3], Beverly Qian Ling Low[1], Yongpeng Liu[1], Ron Milo[3], Erwin Reisner[1,*]

[1] Yusuf Hamied Department of Chemistry, University of Cambridge, Lensfield Road, Cambridge CB2 1EW, UK.
[2] Department of Biochemistry, School of Biological and Behavioural Sciences, Queen Mary University of London, Mile End Road, London E1 4NS, UK.
[3] Department of Plant and Environmental Sciences, Weizmann Institute of Science, Rehovot 7610001, Israel.

[‡] These authors contributed equally.

[*] Corresponding author. E-mail: reisner@ch.cam.ac.uk

Experimental Section

Materials. Sodium bicarbonate ($NaHCO_3$, Puratronic 99.998%, Alfa Aesar), potassium chloride (KCl, 99.999%, ACROS), DL-dithiothreitol (DTT, ≥99.0%, Invitrogen Life Technologies), tris(hydroxymethyl)aminomethane hydrochloride (TRIS-HCl, >99.0%, Sigma-Aldrich), carbonic anhydrase from bovine erythrocytes (≥95%, specific activity ≥3500 W-A units/mg protein, lyophilized powder, stored at 4 °C until use), carbon dioxide gas (BOC UK), $NaH^{13}CO_3$ (98 atom% ^{13}C, 99% (CP), Sigma-Aldrich), $^{13}CO_2$ (99 atom % 13C, <2% 18O, Cambridge Isotope Laboratories), titanium foil (0.25 mm thick, 99.5%, Alfa Aesar), titanium dioxide nanoparticles (Anatase, ThermoScientific), polystyrene beads (750 nm diameter, 2.7% w/v suspension in water, Polysciences Inc.), methanol (ACS reagent, ≥99.8%, Sigma-Aldrich), Parafilm (Sigma-Aldrich), Zn (dust, 98+%, ACROS), hydrochloric acid (HCl, fuming, 36.5−38%, Honeywell), poly[4,8-bis(5-(2-ethylhexyl)thiophen-2-yl)benzo[1,2-b;4,5-b′]dithiophene-2,6-diyl-alt-(4-(2-ethylhexyl)-3-fluorothieno[3,4-b]thiophene-)-2-carboxylate-2-6-diyl] (PCE10, 1-Material), 5,5′-[[4,4,9,9-tetrakis(2-ethylhexyl)-4,9-dihydro-s-indaceno[1,2-b:5,6-b']dithiophene-2,7-diyl]bis(2,1,3-benzothiadiazole-7,4-diylmethylidyne)]bis[3-ethyl-2-thioxo-4-thiazolidinone] (EH-IDTBR, 1-Material), poly(3,4-ethylenedioxythiophene)-poly(styrenesulfonate) (PEDOT:PSS, Clevios P VP AI 4083, Heraeus), zinc oxide nanoparticles (−3.9 eV work function, Avantama), chlorobenzene (extra dry over molecular sieves ≥99.5%, ACROS), Araldite Standard 2-part epoxy, Araldite 5-Minute Rapid 2-part epoxy, graphite powder (<20 µm, synthetic, Sigma-Aldrich), Nafion ion-exchange membrane (Sigma-Aldrich) and deuterium oxide (99.9 atom % D, contains 0.75 wt% 3-(trimethylsilyl)propionic-2,2,3,3-d_4 acid sodium salt, Sigma-Aldrich) were used as received without further purification. Ultrapure deionized water (Simplicity UV MilliQ system, 18.2 MΩ cm) was used in all experiments.

Preparation of bacteria growth media. M9 minimal medium was prepared using a base solution containing Na_2HPO_4 (6 g/L), KH_2PO_4 (3 g/L), NaCl (0.5 g/L), and NH_4Cl (1 g/L). A separate 100X trace elements solution was prepared by dissolving 5 g of EDTA in 800 mL of water, after which the pH was adjusted to 7.5 with NaOH. Following this adjustment, 498 mg $FeCl_3$ (anhydrous), 84 mg $ZnCl_2$, 765 µL of 0.1 M $CuCl_2$•$2H_2O$, 210 µL of 0.2 M $CoCl_2$•$6H_2O$, 1.6 mL of 0.1 M H_3BO_3, and 8.1 µL of 1 M $MnCl_2$•$4H_2O$ were added. The final volume of this stock solution was brought to 1 L with water and sterilized by filtration through a 0.22 µm filter. The final M9 medium was assembled by supplementing the base solution with 1 mL/L of 0.1M $CaCl_2$, 1 mL/L of 1 M $MgSO_4$, and 10 mL/L of the 100X trace elements solution, resulting in a final pH of 7.1-7.2. In addition, 0-60 mM sodium formate was also supplied into the media as energy source. All the chemicals above are purchased from Sigma-Aldrich.

Bacteria growth. Bacterial cultures were incubated at 37 °C with orbital shaking at 150 rpm under a controlled atmosphere of 5% O_2, 10% CO_2, and balanced N_2. Inoculations were performed using a 1:10 dilution from the parent culture. For adaptation experiments, the parent culture was directly injected into the fresh medium. For all other experiments, cells were harvested and washed prior to inoculation to remove residual components. Specifically, the parent culture was centrifuged at 8000 rpm for 5 min at 4 °C, and the pellet was washed three times with fresh medium before inoculation.

Sequencing. DNA extraction and library preparation procedures were carried out and sequenced by GENEWIZ. A reference genome was constructed based on the *E. coli* strain BW25113 (ref. 1) and the sequence of the CBB enzymes encoding plasmid as a second contig. Sequence alignment and variant calling were achieved by using the breseq pipeline.[2] The breseq program was used to identify genomic variants,

including SNPs and insertion-deletion polymorphisms (INDELs). We note that our analysis does not capture certain types of mutations, such as copy number variations.

Reversion of the *pitA* Mutation. Mutational reversion of the *pitA* allele in the evolved autotrophic strain (transfer #27) was performed by P1 phage-mediated transduction following established protocols.[3] A P1 lysate was prepared from donor strain JW5678-2 of the Keio collection,[4] which carries a *yrhC*::*kan* resistance cassette located adjacent to the *pitA* locus. Transductants were selected by plating on LB agar supplemented with kanamycin as a selection marker. Genotypes of kanamycin-resistant colonies were verified by multiple allele-specific colony PCR (MASC) using primers designed to distinguish the wild-type and mutant *pitA* alleles.[5] Primer sequences were as follows: mutant-specific forward, 5′-CCTTCCGGCTGCGGTACTT-3′; wild-type–specific forward, 5′-CCTTCCGGCTGCGGTACTG-3′; and reverse, 5′-GCGCGTTGTTAGTGGTACTGC-3′. For growth experiments, a control strain retaining the evolved *pitA* mutation while carrying the linked *yrhC*::*kan* marker was included to account for potential effects of the antibiotic cassette.

Growth in plate-reader. The growth test experiments were conducted in 96 well-plates. The final volume of each well was 200 µL (50 µL of mineral oil and 150 µL culture). The media consisted of M9 media supplemented with 40mM formate, and trace elements (without addition of vitamin B1). Bacterial cells were seeded from a culture tube. Growth temperature was set to 37 °C, and either aerated with ambient air or air with elevated CO_2 (10%) either with ambient or reduced oxygen (5%). OD_{600} measurements were taken every 12 min using a Biotek Synergy H1 plate reader.

Purification of FDH for electrochemistry and photoelectrochemistry (PEC). Stock solutions of [W]-FDH from *Nitratidesulfovibrio vulgaris* Hildenborough (*Nv*H) were stored at −40 °C in an anaerobic glovebox (MBraun, N_2 atmosphere). Each Eppendorf tube as received contained 10 µL of 50 µM FDH in 20 mM TRIS-HCl, 10 % v/v glycerol and 10 mM $NaNO_3$ (pH 7.6). The FDH was thawed immediately prior to purification, washed 3 rounds with 20 mM TRIS-HCl (i.e. no glycerol, no $NaNO_3$) to remove most of the glycerol content and re-concentrated via ultra-centrifugation (Amicon Ultra-Centrifugal Filters, 0.5 mL, 30 kDa MWCO). All FDH stocks were purified fresh prior to each electrochemical or PEC experiment and not re-frozen again for storage.

Fabrication of inverse opal TiO_2 (IO-TiO_2) electrodes. The IO-TiO_2 electrodes were fabricated as previously reported.[6,7] In brief, titanium foil (1 cm × 2 cm for electrochemistry, 1.2 cm × 0.7 cm for PEC) was thoroughly cleaned by sonication in ethanol for 30 minutes, followed by attachment of a Parafilm ring template (defining an electrode area of 0.19 cm^2). A polystyrene bead suspension (1 mL, as received from Polysciences Inc.) was centrifuged at 10000 r.p.m. to remove the supernatant and re-dispersed in MeOH (1 mL). After re-centrifugation at 10000 r.p.m., MeOH was removed and TiO_2 nanoparticles (30 mg in H_2O/MeOH mixture, 4:1 volume ratio, 300 µL) were added. The mixture was sonicated for 5 min in ice cold water (< 5 °C) before drop-casting onto the titanium foil. The electrodes were then left to dry for an hour before annealing in an oven at 500 °C for 20 min (1 °C min^{-1} ramp rate).

Fabrication of organic photovoltaics (OPVs). Conventional architecture OPVs based on the bulk heterojunction PCE10:EH-IDTBR were fabricated as reported.[8] Briefly, patterned ITO-coated glass substrates (1.3 × 1.3 cm^2) were sonicated in acetone, ethanol, isopropyl alcohol and deionized water. The cleaned substrates were then subjected to UV-Ozone treatment for 40 min (BioForce Nanosciences UV/Ozone ProCleaner). PEDOT:PSS was filtered through a Millex-GP 0.22 µm PES filter, spin-coated at 4000 r.p.m. and annealed on a hot plate at 383 K for 40 minutes. A solution of PCE10:EH-IDTBR in anhydrous chlorobenzene (1:2 weight ratio, 24 mg ml^{-1}) was then spin-coated at 3000 r.p.m., followed by ZnO nanoparticles at 4000

r.p.m. Finally, Ag (100 nm) was thermally evaporated through a custom-made patterned mask under vacuum (~10^{-5} mbar), defining an active area of ~0.5 × 0.5 cm^2 for each device.

OPV characterization. A Newport Oriel 67005 solar light simulator equipped with an AM 1.5G optical filter was calibrated to 1 sun (100 mW cm^{-2}) using a standardized open Silicon solar cell (ReRa Solutions, Device Code: RQN211399, Reference: 211300, Area: 4 cm^2). Reverse and forward *J-V* sweeps of the OPVs were measured at a scan rate of 100 mV s^{-1} over 20 mV steps from −0.1 V and 1.1 V using an Ivium CompactStat potentiostat. No additional masking of the devices was required and each active area (~0.5 × 0.5 cm^2) was measured manually.

Electrochemistry and PEC (without bacteria in electrolyte). The IO-TiO_2 electrodes were masked with Kapton tape to define an active area of 0.19 cm^2, followed by wiring to metal rods with copper tape. Parafilm and Teflon tape were further added to prevent water infiltration. In a N_2-filled glovebox, purified FDH (500 pmol) was activated with DTT for 20 minutes and drop-casted onto the IO-TiO_2 electrode. Carbonic anhydrase (from bovine erythrocytes, 100 pmol) was then drop-cast onto the same electrode and left to incubate for a few min before full immersion in solution.

An H-cell separated by a Nafion membrane was used, with 5 mL of 50 mM $NaHCO_3$ + 50 mM KCl (CO_2-purged, pH 6.45) in both cathodic and anodic compartments (**Figure S10**). This volume was the minimum required to fully wet the membrane and ensured the maximum possible formate concentration for accelerated microbial growth. A three-electrode configuration was adopted, with an IO-TiO_2|FDH+CA working electrode, a Ag/AgCl reference electrode (in saturated NaCl, BASi MW-2030) and a platinum mesh counter electrode. After purging the H-cell with CO_2 prior to each experiment, protein film voltammetry (PFV) scans (5 mV s^{-1}) were then recorded from −0.4 to +0.2 V vs. RHE on an Ivium CompactStat potentiostat, followed by controlled potential electrolysis (CPE) at −0.4 V vs. RHE for 10 h at room temperature with continuous stirring. Current density values were calculated based on the electrode's geometric surface area of 0.19 cm^2. Potentials measured against Ag/AgCl were converted to the RHE scale using Equation 1 (at 298 K):

$$E_{(\text{V vs. RHE})} = E_{(\text{V vs. Ag/AgCl})} + 0.059 \times \text{pH} + 0.197\ \text{V}. \qquad \text{(Equation 1)}$$

The formate-containing electrolyte was then transferred into a glass vial, filter-sterilized and supplemented with trace elements for subsequent inoculation with the washed *E. coli* strain (#27). The vial was then purged with a gas mixture containing 10% CO_2, 85% N_2, and 5% O_2 to support autotrophic growth.

A similar procedure was adopted for PEC with organic photoelectrodes. OPVs were interfaced with an IO-TiO_2 electrode using conductive graphite epoxy paste, followed by wiring and more epoxy encapsulation to prevent moisture infiltration.[8] Purified FDH (500 pmol) and carbonic anhydrase (100 pmol) were then co-immobilized onto the electrode. A Newport Oriel 67005 solar light simulator (AM 1.5G, 100 mW cm^{-2}) was calibrated to 1 sun using a certified Newport 843-R optical power meter. PFV scans were recorded between −0.1 V vs. RHE and 1.1 V vs. RHE at a scan rate of 10 mV s^{-1} and CPE experiments were conducted at 0.6 V vs. RHE under chopped illumination (cycles of 50 min light on, 10 min light off). Photocurrent density values were calculated based on a device active area of 0.25 cm^2.

Assembly of one-pot $BiVO_4$||OPV|IO-TiO_2|FDH+CA-microbe domino cascade. Tandem devices with $BiVO_4$ were constructed by wiring the OPV photocathodes to $BiVO_4$ photoanodes in a back-to-back configuration (defined active area of ~0.5 × 0.5 cm^2). The $BiVO_4$ photoanodes with a TiCo O_2 evolution catalyst were prepared as previously reported.[9,10] In a N_2-filled glovebox, 500 pmol of purified FDH and 100 pmol of carbonic anhydrase were immobilized onto IO-TiO_2 and the semi-artificial leaf was immersed in a CO_2-saturated, 50 mM $NaHCO_3$ + 50 mM KCl buffer solution (5 mL). This buffer solution also contained *E. coli* with an optical density (OD_{600}) of ~0.01. A one-compartment jacketed glass reactor was used. After

purging the reactor with CO_2, bias-free CPE was performed under 1 sun illumination (AM1.5G, 100 mW cm^{-2}, with a UV filter allowing $\lambda > 400$ nm) at 37 °C with continuous stirring for 20 h. The reactor was subsequently supplemented with trace minerals and re-purged with 10% CO_2, 85% N_2 and 5% O_2, before being transferred to a stationary incubator at 37 °C for 2 more days (i.e. ~3 days in total). A control experiment was conducted in parallel, comprising of *E. coli* inoculated at an OD_{600} of ~0.01 and kept in the dark incubator for 3 days. After the experiments, SEM imaging of the IO-TiO_2 and $BiVO_4$ (photo)electrodes were performed, followed by energy-dispersive X-ray (EDX) mapping to check for salt deposition.

Product quantification. Optical density (OD) measurements at 600 nm were performed on a Cary 60 UV-vis spectrometer. ΔOD_{600} refers to the difference in OD measured at the start and end of the experiment. Formate and acetate production by the microbes over time, as well as the glycerol amounts post-purification, were determined by quantitative ^{1}H NMR (400 MHz, D_2O) using 3-(Trimethylsilyl)propionic-2,2,3,3-d_4 acid sodium salt as an internal standard.[11] Any microbe-containing electrolyte was filtered using a 0.2 µm Millipore filter before NMR measurements. Formate Faradaic yields (FY) for electrochemistry and PEC experiments were calculated using Equation 2:

$$FY\ (\%) = \frac{nZF}{Q} \times 100 \qquad \text{(Equation 2)}$$

where *n* is the number of moles of product, Z is the number of electrons needed per molecule of product (Z = 2 for formate production and Z = 4 for oxygen production), F is the Faraday constant (96485 C mol^{-1}), and Q is the total charge passed. The total charge Q was determined by integrating the current trace over a defined period. TON and TOF values for formate production were calculated based on the amount of enzyme drop-cast onto the IO-TiO_2 electrodes. Oxygen evolution was monitored using a NeoFox-GT fluorometer and Fospor-R fluorescence oxygen sensor probe (Ocean 584 Optics).

Materials characterization. Field emission scanning electron microscope imaging (TESCAN MIRA3 FEG-SEM) was used to study the hierarchically porous morphology of the IO-TiO_2 electrodes. The electron beam accelerating voltage was 5 kV and both Axial (In-column axial BSE/SE detector) and MD (In-column Energy-Filtered Multidetector) detectors were used. SEM images of the $BiVO_4$ photoanode post-microbial growth were acquired in a similar manner. For more in-depth analysis of the surface topography, the $BiVO_4$ sample was coated with carbon and imaged with LSE (low-energy backscattered electron; motorized) and Everhart-Thornley (secondary electron) detectors.

Statistical Analysis. All experiments regarding bioelectrochemical formate synthesis, utilization, and solar-driven autotrophic growth were performed in triplicates (n=3). Data are presented as the mean ± standard deviation (SD) unless otherwise noted.

Supplementary Notes

Supplementary Note 1 regarding glycerol from FDH stocks serving as an alternative carbon source and its removal to ensure autotrophic growth.

The [W]-formate dehydrogenase (FDH) used in this study was purified and stored in a buffer containing 10% (v/v) glycerol (20 mM TRIS-HCl, 10 mM $NaNO_3$, pH 7.6).[12,13] Although glycerol is required as a stabilizing agent and cryoprotectant,[14,15] it can also serve as an alternative carbon source for *E. coli*, potentially confounding assessments of engineered autotrophic growth. We therefore established and validated a protocol to remove this contaminant prior to experiments.

We first confirmed that glycerol consumption by *E. coli* was a confounding factor in our system (**Figure S3, S4**). Cells were cultured with formate generated either by an OPV|IO−TiO_2|FDH+CA photocathode (yielding 11 mM) or a bias-free tandem device with $BiVO_4$ (yielding 5 mM). While initial growth over two days correlated with formate consumption (concentration decreased from 11 mM to 8 mM) and a corresponding increase in cell density (OD_{600} from 0.02 to 0.04), ^1H qNMR analysis revealed a subsequent metabolic shift. Between days 2 and 4, the rate of formate consumption slowed (**Figure S4A**) as the cells began to metabolize residual glycerol from the FDH stock, evidenced by the disappearance of its characteristic NMR peaks (**Figure S4E**).

To eliminate this heterotrophic growth pathway, we developed a washing procedure based on ultracentrifugation and evaluated filters with two different molecular weight cut-offs (MWCOs). Three washing cycles using a 50 kDa MWCO filter successfully reduced the initial glycerol content 24-fold (from 12 µmol to 0.5 µmol) (**Figure S5**). However, this protocol led to a major loss of electrochemical activity, attributed to enzyme (MW: 141.44 kDa, PDB: 6SDR) loss through the filter membrane. Conversely, a single wash step with a smaller 30 kDa MWCO filter retained more FDH, enabling a high current density of −3.2 mA cm^{-2} at −0.4 V vs. RHE and the production of 17.2 mM formate (99.6% FY) over 7 hours (**Figure S6**). However, this abbreviated procedure only provided a 6-fold reduction in glycerol content, leaving 2 µmol in the final solution.

Therefore, the optimized protocol involved three washing and re-concentration cycles using a 30 kDa filter with 20 mM TRIS-HCl. This effectively removed glycerol while retaining FDH for biohybrid experiments, thereby ensuring that cell growth is strictly dependent on the electrochemically and PEC generated formate (**Figure S7-S13**, also see Experimental Section above).

Supplementary Note 2 regarding oxygen limitation-triggered acetate production.

We observed that hypoxic conditions (O_2 < 3%) severely inhibited bacterial growth and concurrently led to the accumulation of acetate (6–11 μM) in the medium (**Figure S15**). Acetate also accumulated during semi-artificial leaf integration experiments, reaching 0.12 mM by day 2.5 in the presence of trace elements (**Figure S23**). Crucially, this process was reversible: upon replenishing the headspace with sufficient O_2 and incubating for an additional day, acetate was consumed, accompanied by a corresponding increase in bacterial OD_{600}. As formate was almost entirely depleted by day 2.5, these results suggested that O_2 limitation prompts a metabolic shift. Instead of contributing to biomass or entering the TCA cycle, fixed carbon was channeled into a fermentative pathway, resulting in acetate synthesis (**Figure S28**).

To confirm that this acetate was derived from newly fixed carbon, we performed an isotope labelling experiment, culturing *E. coli* with ^{13}C-labelled HCO_3^- and ^{13}C-labelled formate under a headspace containing 0–5% O_2 (balanced with N_2). After two days of incubation, ^{13}C-labelled acetate was identified in cultures supplied with 1.1% and 2.4% O_2 (**Figure S29**). A background signal from ^{12}C-acetate, likely originating from the previous culture with ^{12}C inoculum, was detected in all samples. No ^{13}C-acetate was detected under complete anoxia (0% O_2). After another eleven days of incubation (in total thirteen days), more ^{13}C-labelled acetate was identified in the 1.1% and 2.4% O_2 group; especially the 2.4% O_2, the ^{13}C-labelled acetate increased from 0.01 mM (2 days) to 0.23 mM (13 days). This demonstrates that acetate production arises from the fixation of external ^{13}C sources (HCO_3^- or formate) and not from the catabolism of internal carbon stocks or dead cells. The absence of O_2 correlated with low biomass and reduced ^{13}C-formate consumption, corroborating our initial findings (**Figure S16**). Furthermore, our observation that oxygen concentration shifts carbon flux between biomass and acetate (**Figure S29**) suggests a powerful strategy for future metabolic engineering. While we prioritized biomass in this work, one could deliberately leverage and engineer this hypoxic switch to channel fixed carbon into a diverse portfolio of valuable bioproducts beyond simple growth.

Supplementary Note 3 regarding the design rationale for one-pot integration of abiotic and biotic components.

Realising autonomous, solar-driven CO_2-to-biomass conversion in a single reactor requires the simultaneous resolution of three physicochemical compatibility barriers between the abiotic photoelectrochemical components and the biotic *E. coli* chassis. Prior biohybrid platforms have addressed each barrier in isolation, typically by physically separating the abiotic and biotic compartments (**Table S1**). The barriers, and the corresponding design choices adopted in this work, are summarized below.

(i) Kinetic matching of electron flux to microbial uptake. The rate of electrochemical formate generation at the photocathode must align with the metabolic uptake rate of the microbial chassis. If formate is produced faster than it is consumed, it accumulates, lowers the local pH and reduces FDH activity; if produced too slowly, the bacteria starve. To address this, an enzyme cascade ([W]-FDH and CA) was selected over metallic CO_2-reduction electrocatalysts. The cascade delivers ~100% formate selectivity at near-thermodynamic potentials, a unique advantage of FDH over synthetic CO_2 reduction catalysts that typically co-generate CO and other byproducts potentially toxic to *E. coli*. The cascade also produces a soluble, diffusive energy vector (formate) whose turnover at the photocathode can be tuned by light intensity, applied potential, and enzyme loading to fall within the bacterial uptake window observed in the dose-response data (**Figure S14**).

(ii) Chemical non-toxicity of materials and intermediates. Leached metal ions from inorganic semiconductors and CO_2 reduction electrocatalysts, sacrificial electron donors, and UV-generated reactive species are cytotoxic and incompatible with sustained microbial growth. To address this, organic semiconductors (PCE10:EH-IDTBR bulk heterojunction) and graphite epoxy encapsulants were selected over metal halide perovskites, metal chalcogenides, and metal-alloy encapsulants. The OPV stack avoids the leaching of toxic metal ions into the electrolyte, and its tunable visible-light absorption enables bias-free operation under UV-filtered illumination ($\lambda > 400$ nm) that does not damage the bacteria.

(iii) Shared microenvironment for water oxidation, FDH catalysis, and bacterial growth. The same electrolyte must simultaneously support water oxidation at the $BiVO_4$ photoanode, FDH activity at the IO-TiO_2 photocathode, and *E. coli* growth in bulk solution. The conflicting requirements include trace-metal nutrients that are essential for microbial growth but may inhibit electrocatalysis (**Figure S15**), and localised O_2 that is needed for aerobic respiration but could deactivate FDH. To address this, a 50 mM $NaHCO_3$ / 50 mM KCl electrolyte was used in place of the Good's buffers (e.g., TRIS, MES, MOPS) or sacrificial reagents commonly adopted in prior biohybrid systems (**Table S1**). This benign electrolyte supports $BiVO_4$ water oxidation, FDH stability at the cathode, and *E. coli* growth in a single solution. Residual incompatibilities, in particular the trace-mineral and O_2 supply mismatches, were managed operationally by sequencing the irradiation phase and the post-irradiation incubation phase (**Figure 5B, Figure S23, Supplementary Note 2**) rather than chemically.

Together, these three design choices enable the direct coupling of a photoelectrochemical enzyme-microbe cascade to a water-oxidising photoanode in a single pot. The semi-artificial leaf functions as an abiotic thylakoid that converts sunlight, CO_2, and H_2O into O_2 and formate; formate, in turn, drives CO_2 fixation into biomass by the evolved *E. coli* strain, mirroring the light and dark phases of natural photosynthesis.

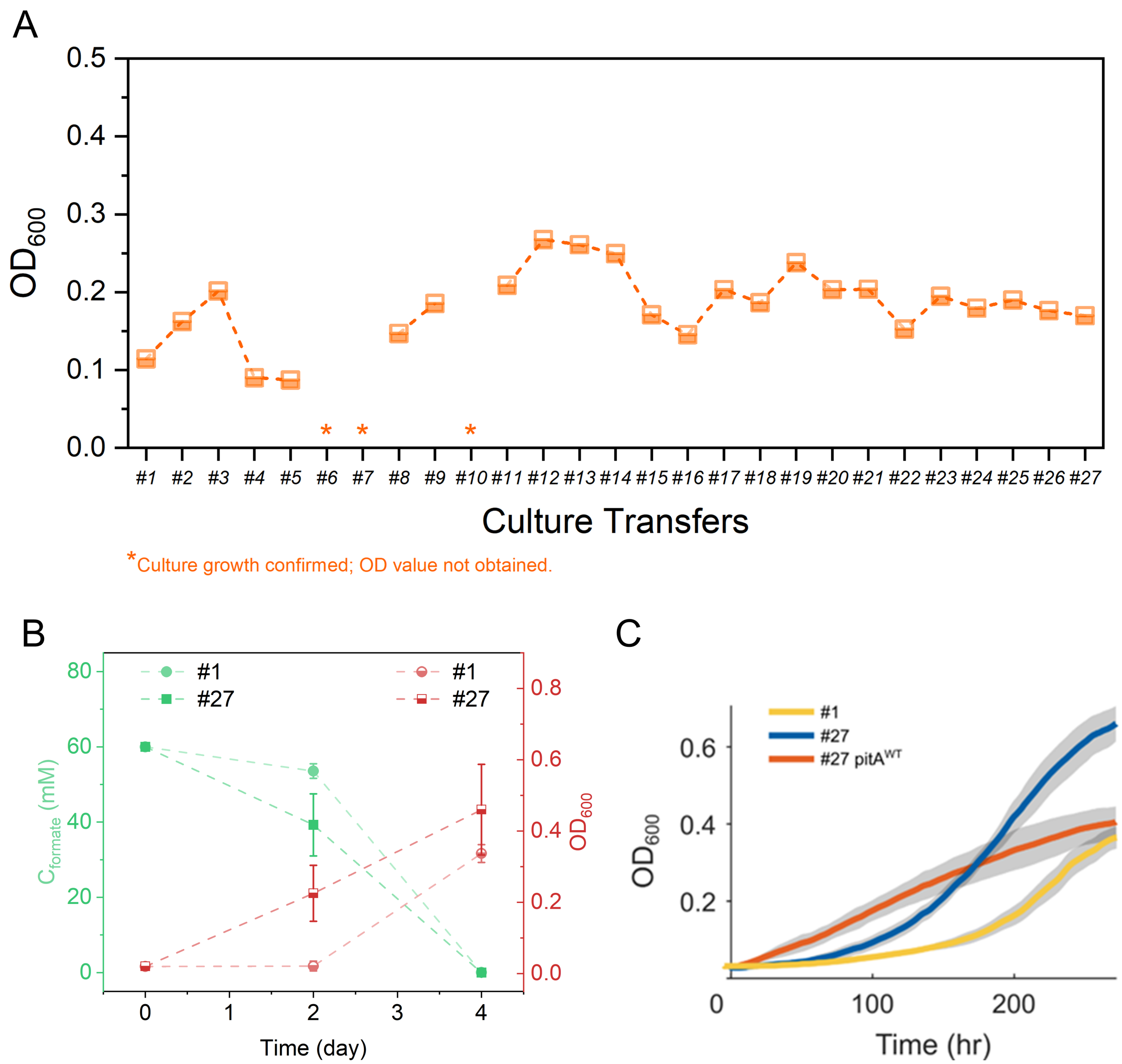

Figure S1. Dynamics of adaptive laboratory evolution on formate. (A) Evolutionary trajectory over 27 serial transfers. Data points represent the final optical density (OD_{600}) reached at the stationary phase of each passage. The asterisk (*) denotes transfers where growth was visually confirmed but OD_{600} was not quantified. (B) Kinetic profiling of growth and formate consumption. Comparative time-course analysis of the parental strain (Transfer #1, circles) versus the evolved strain (Transfer #27, squares) in autotrophic batch culture. The evolved strain displays significantly faster formate oxidation kinetics and a shortened lag phase relative to the parent strain. Data are presented as the mean ± standard deviation from three biological replicates. (C) Functional validation of the *pitA* mutation. Comparative growth analysis of the parental strain (Transfer #1), the evolved strain (Transfer #27), and the evolved strain with a WT allele of *pitA* (#27-pitA^{WT}) in autotrophic batch culture. Growth kinetics were measured using a plate-reader assay, revealing that reversion of *pitA* resulted in reduced fitness compared to the evolved strain, supporting the functional relevance of the mutation.

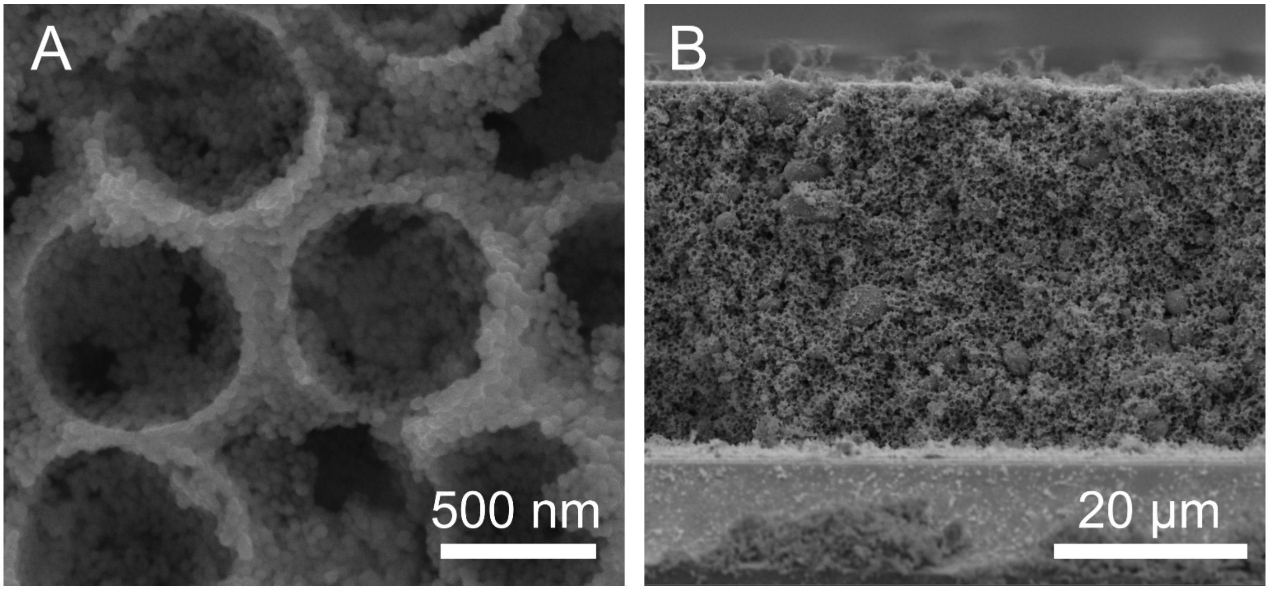

Figure S2. Scanning electron microscopy (SEM) images of the IO-TiO_2 electrodes on Ti foil. (A) Surface analysis recorded with a view field of 2 µm (Detector: In-beam Secondary Electron) and (B) Cross-section imaging recorded with a view field of 64 µm (Detector: Secondary Electron Everhart-Thornley). Both images were recorded using a 5 kV electron beam accelerating voltage. The electrode in (B) was prepared using the same procedure on an ITO-glass substrate, to facilitate sample cutting for SEM imaging.

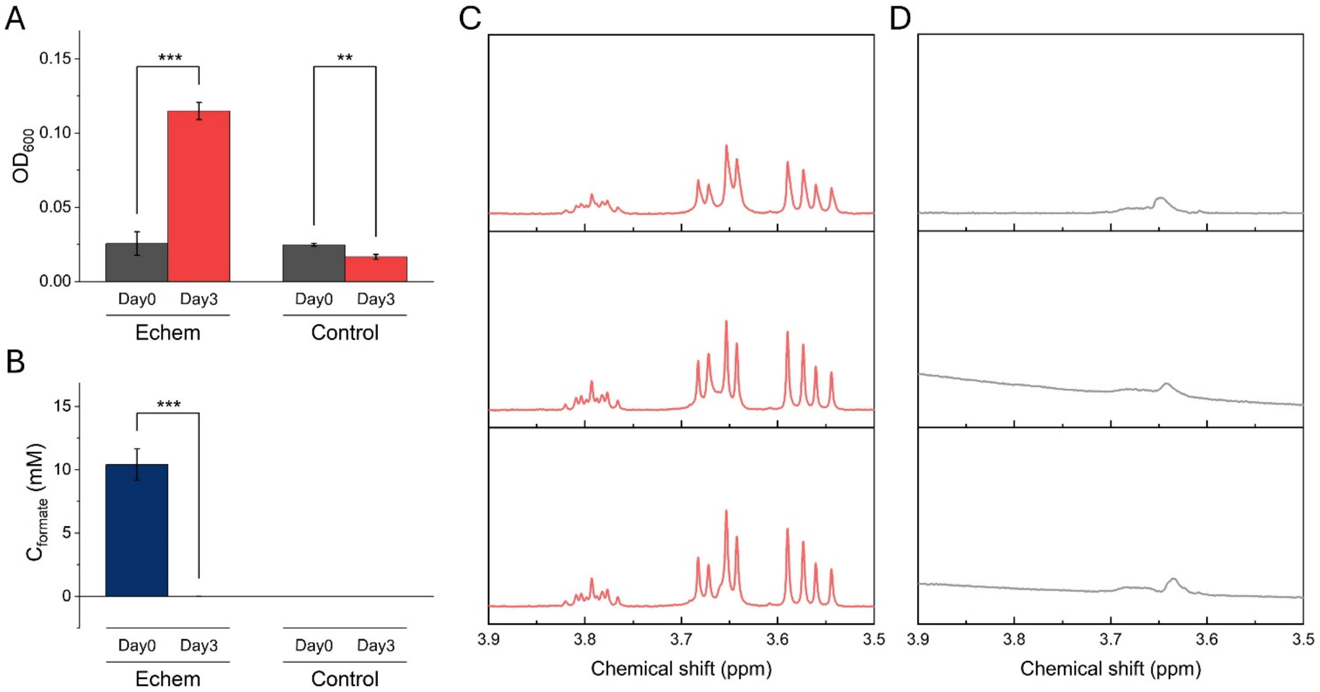

Figure S3. Preliminary test growing *E. coli* with electrogenerated formate using an FDH-immobilized carbon-felt|TiO_2 electrode. Change in (A) OD_{600}, and (B) formate concentration after 3 days of inoculating *E. coli* into formate-containing electrolyte and correspondingly. ^1H NMR spectra of (C) Day 0 and (D) Day 3 samples in the zoomed in area of glycerol peaks. Conditions: The electrochemical (Echem) experiment employed a 3-electrode setup consisting of a previously reported working electrode (carbon-felt|TiO_2|FDH),[13] a Pt counter electrode and an Ag/AgCl (saturated KCl) reference electrode. The electrolyte contained $NaHCO_3$ (100 mM) with KCl (50 mM). CPE was performed at −0.5 V vs. RHE for 24 h. The control experiment used the same electrolyte without any formate.

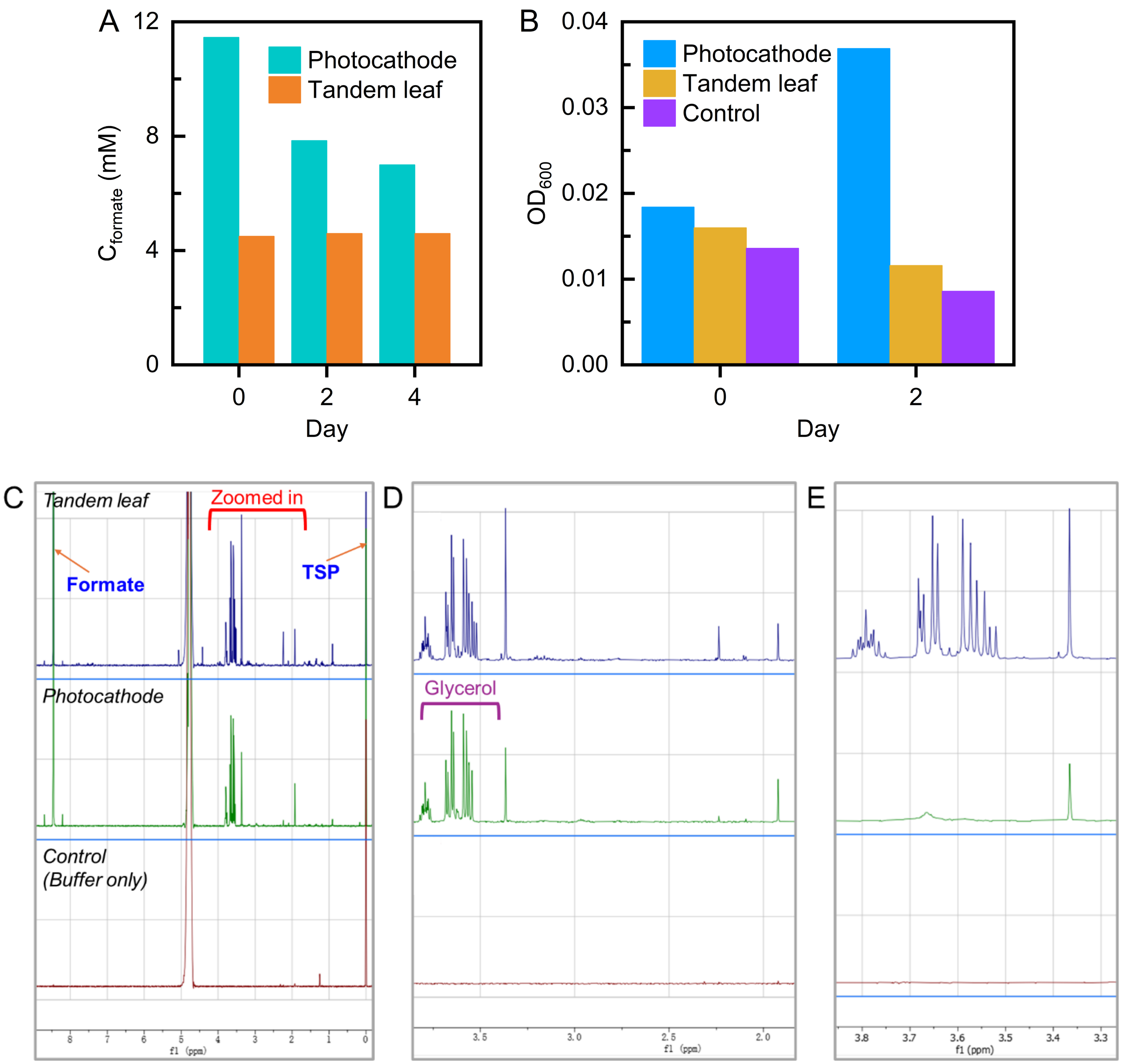

Figure S4. *E. coli* growth switches from PEC-generated formate to glycerol from unpurified FDH. (A) Change in formate concentration over 4 days after incubation with *E. coli* (serum bottle purged with 10% CO_2, 5% O_2 and 85% N_2). (B) OD_{600} of the buffer solution over 2 days. (C-E) ^{1}H NMR spectra (400 MHz, D_2O) of the reaction electrolytes on (C) Day 0 (full range), (D) Day 0 (zoomed in from (C)) and (E) Day 4. PEC CPE with the photocathode (OPV|IO-TiO_2|FDH+CA) was performed at 0.6 V vs. RHE for 10 h, while that of the tandem device ($BiVO_4$||OPV|IO-TiO_2|FDH+CA) was conducted under bias-free conditions for 24 h. Conditions: 500 pmol of FDH and 100 pmol of CA, 50 mM $NaHCO_3$ + 50 mM KCl, room temperature, with stirring.

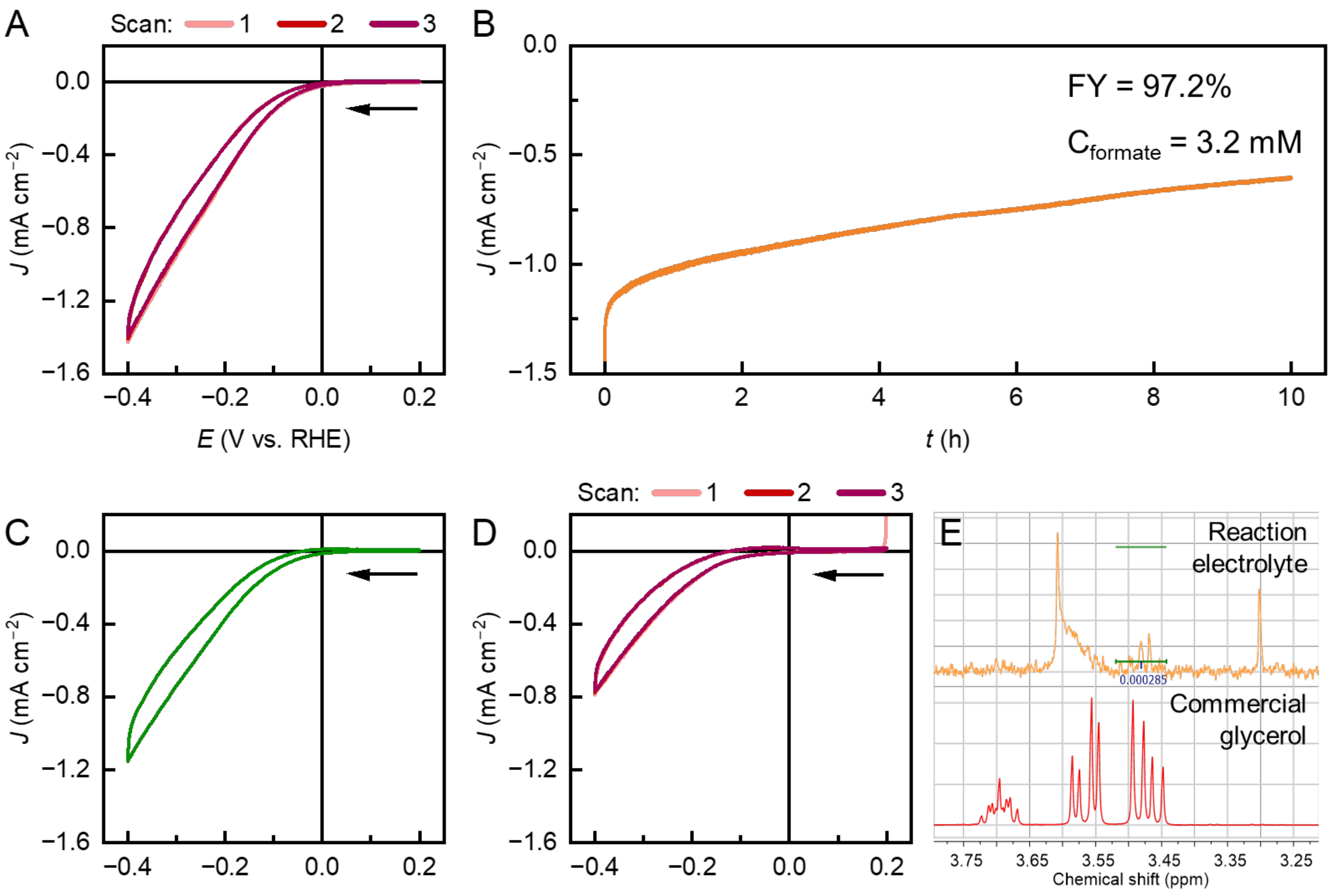

Figure S5. Electrochemistry with purified FDH on IO-TiO_2 electrodes (50 kDa filter). (A) PFV scans of the washed FDH immobilized on IO-TiO_2 recorded at 5 mV s^{-1} before 10 h CPE. 3 consecutive scans were recorded. (B) CPE trace recorded over 10 h at −0.4 V vs. RHE. (C) PFV scan of pristine FDH (100 pmol, without purification) immobilized on IO-TiO_2 for direct comparison with (A). (D) PFV scans recorded after 10 h CPE. (E) ^{1}H NMR spectrum of the reaction electrolyte as compared to commercial glycerol (400 MHz, D_2O with TSP as internal standard). Integrations were performed with respect to the TSP standard (normalized area of 1). Conditions: 100 pmol purified FDH only (no CA), 50 mM $NaHCO_3$ + 50 mM KCl, room temperature, with stirring.

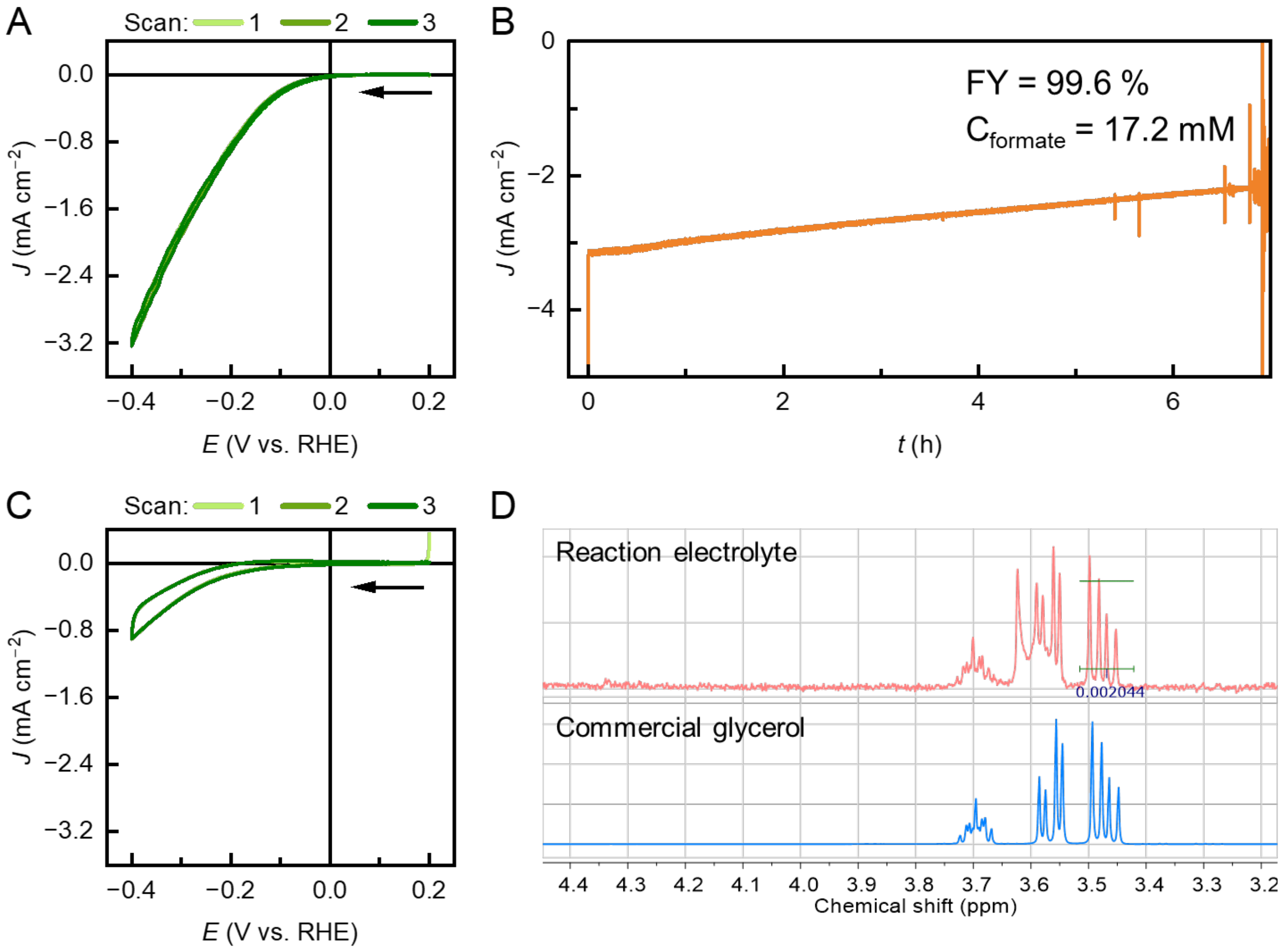

Figure S6. Electrochemistry with purified FDH on IO-TiO_2 electrodes (30 kDa filter). (A) PFV scans recorded at 5 mV s^{-1} before 10 h CPE. (B) CPE trace recorded over 7 h at −0.4 V vs. RHE. The noisy trace towards the end was attributed to electrical connection issues with the potentiostat. (C) PFV scans recorded after 10 h CPE. (D) ^{1}H NMR spectrum of the reaction electrolyte (400 MHz, D_2O with TSP as internal standard). Integrations were performed with respect to the TSP standard (normalized area of 1). Conditions: 500 pmol purified FDH + 100 pmol CA, 50 mM $NaHCO_3$ + 50 mM KCl, room temperature, with stirring. The current densities in (A) are consistent with those previously reported for 500 pmol pristine FDH + 100 pmol CA,[16] indicating minimal enzyme losses by using a 30 kDa filter as compared to one with a larger MWCO (**Figure S5A, C**). Glycerol is still present in (D) after one round of washing.

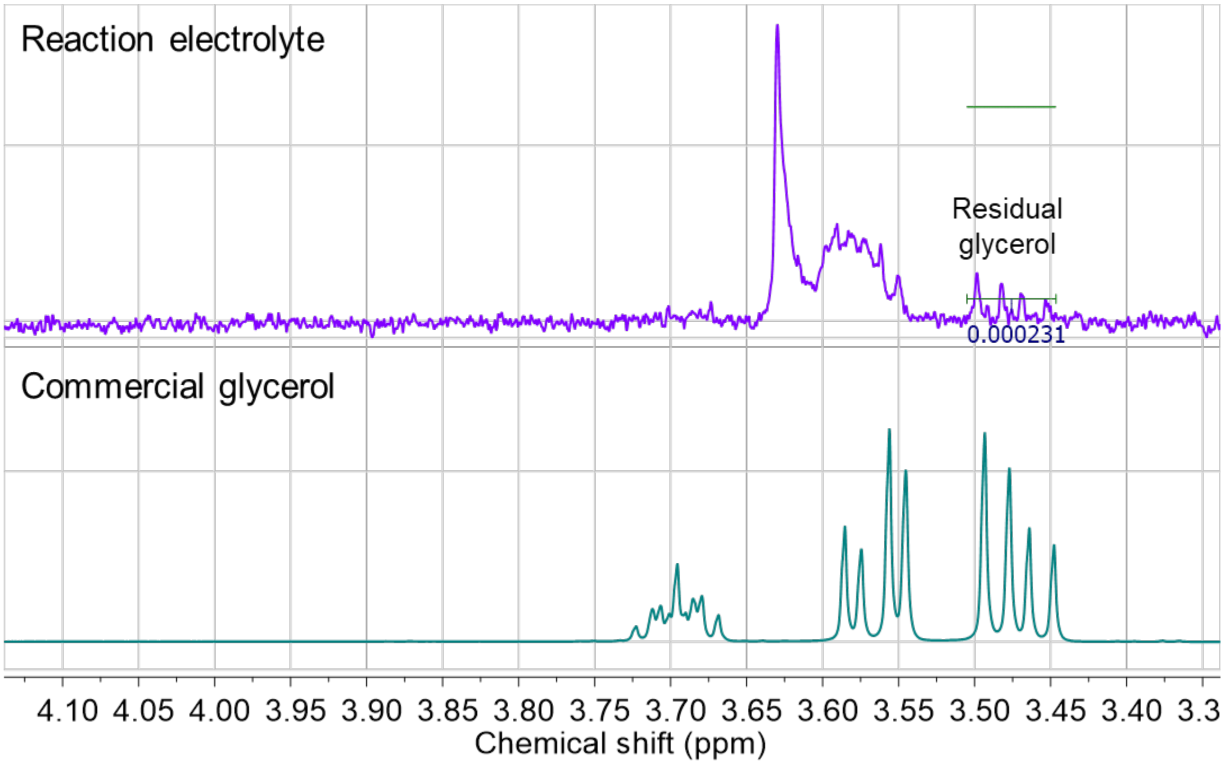

Figure S7. ^1H NMR spectrum (400 MHz, D_2O, delay time = 60 s) of the reaction electrolyte post-CPE. FDH (500 pmol) was purified using the optimized washing protocol. The ^1H NMR spectrum for commercial glycerol is included for comparison. The peak at ~3.63 ppm can be attributed to TRIS-HCl (see **Figure S8C**).

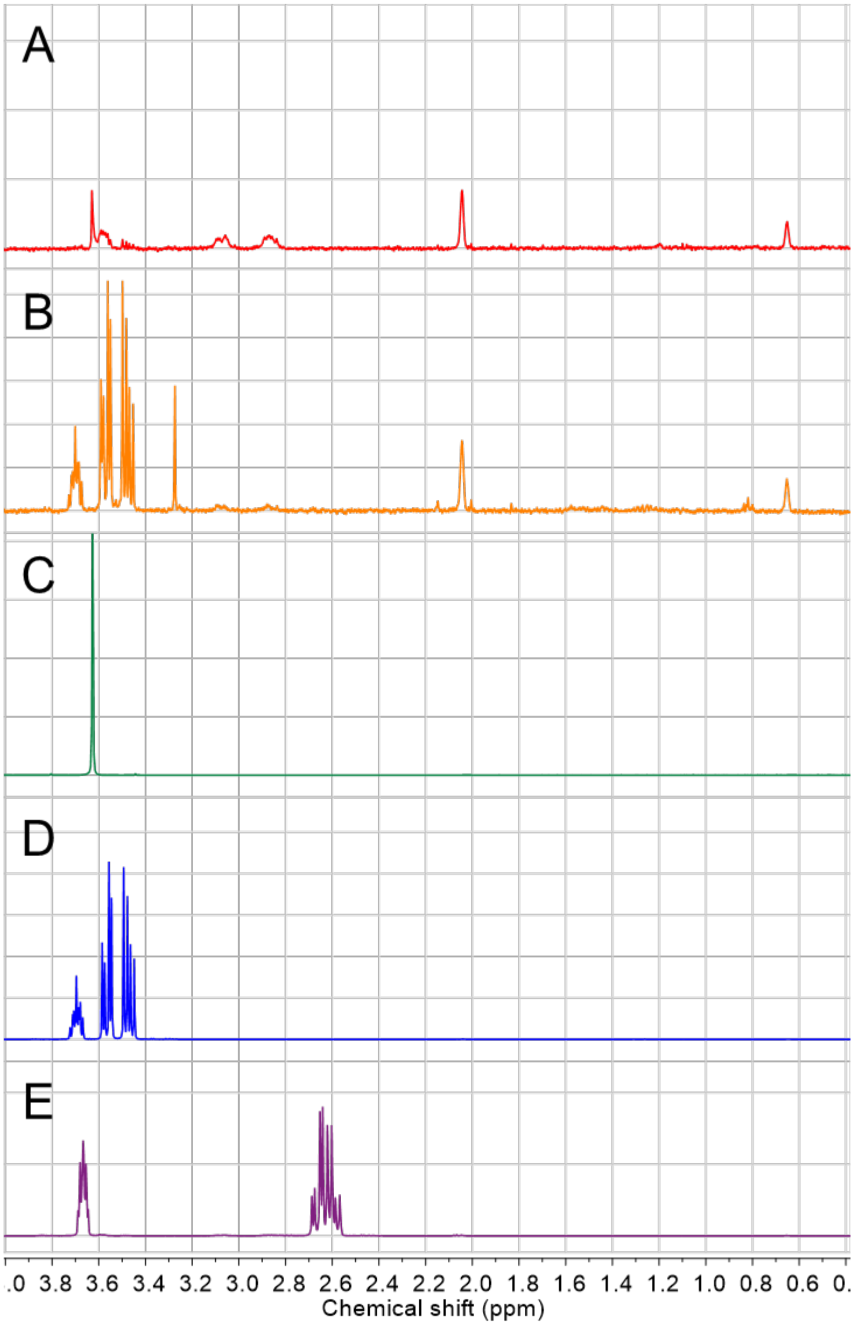

Figure S8. ^{1}H NMR spectra of various electrolyte components. (A-B) Electrolyte after CPE with (A) purified FDH and (B) pristine FDH. (C-E) Commercial samples of (C) TRIS-HCl, (D) glycerol and (E) DTT. The spectra were collected on a 400 MHz spectrometer (D_2O with TSP internal standard). Delay time = 60 s.

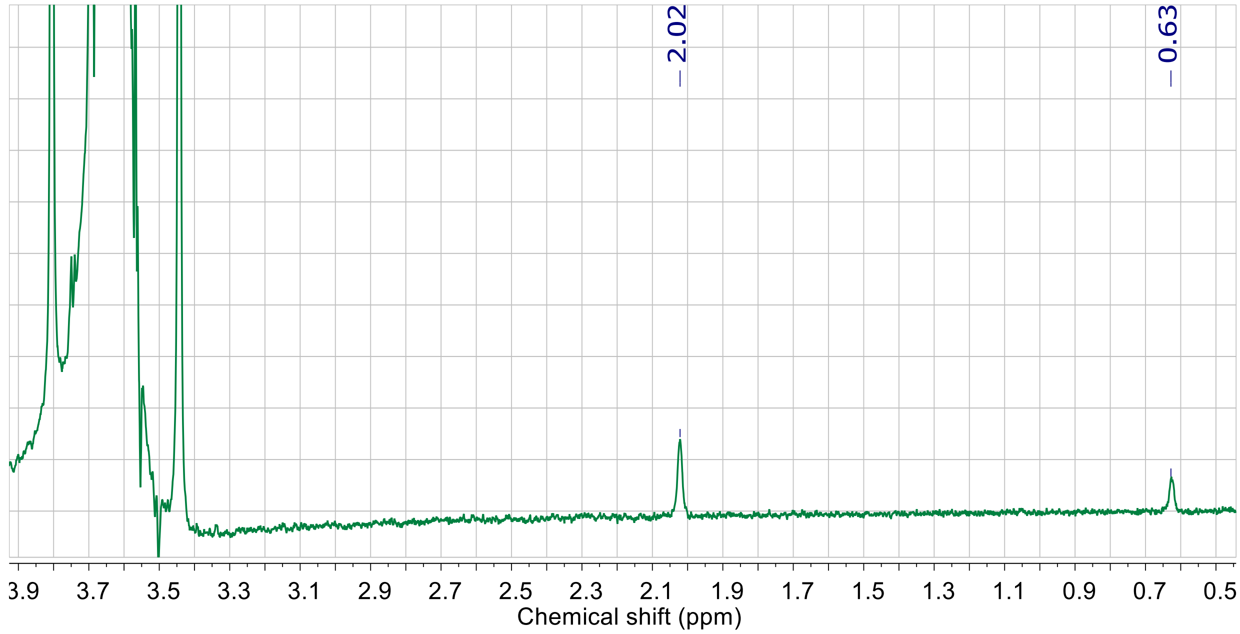

Figure S9. ^{1}H NMR spectra of TRIS-HCl. These two peaks not seen in **Figure S8C** are visible upon zooming in.

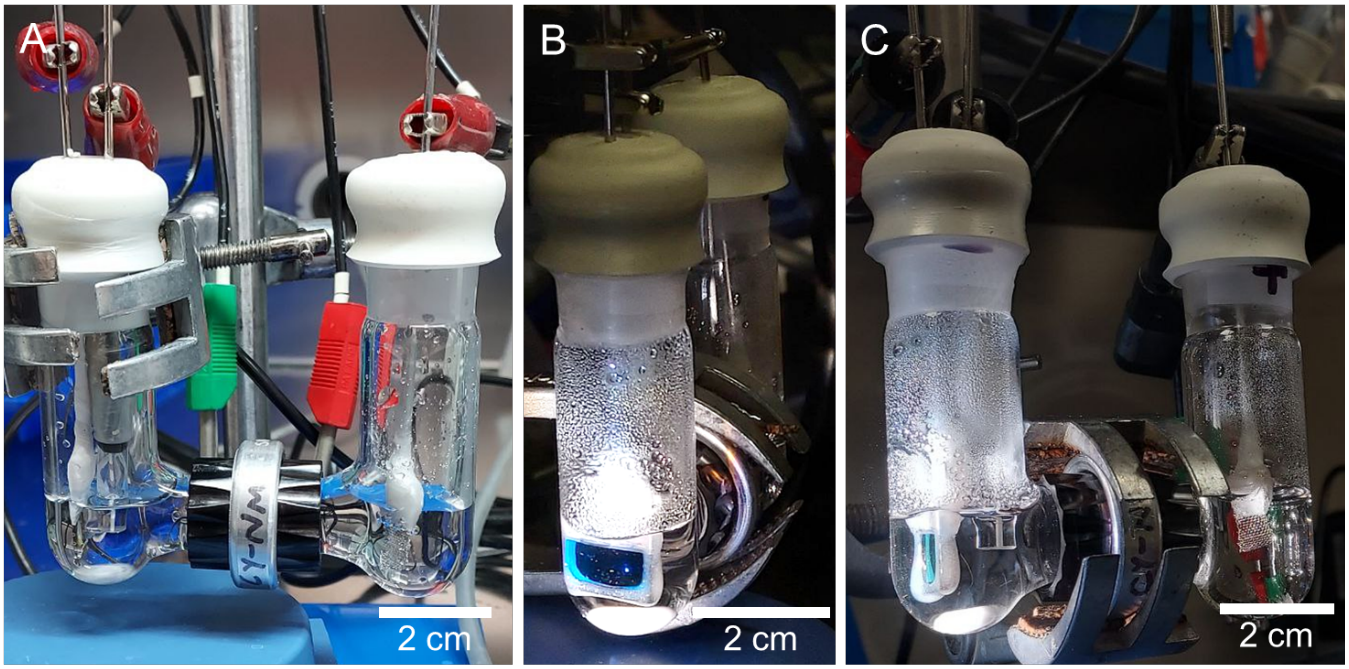

Figure S10. Photographs of H-cells used for both electrochemical and PEC formate generation. (A) Electrochemistry with IO-TiO_2|FDH+CA. (B-C) PEC with OPV|IO-TiO_2|FDH+CA from the (B) front view (C) side view. Conditions: 500 pmol purified FDH+CA, 50 mM $NaHCO_3$ + 50 mM KCl (pH 6.45, CO_2-purged, room temperature), with stirring. The same reactor design was kept for both electrochemical and PEC experiments to ensure consistency. Both cathodic and anodic compartments were each filled with 5 mL of electrolyte and separated by a Nafion membrane located in the middle of the black Torion glass joint. 5 mL is the minimum volume required to ensure full wettability of the Nafion membrane. PEC experiments were performed under 1 sun irradiation (AM1.5G, 100 mW cm^{-2}). The blue hue in (B) and (C) are from the organic bulk heterojunction PCE10:EH-IDTBR.

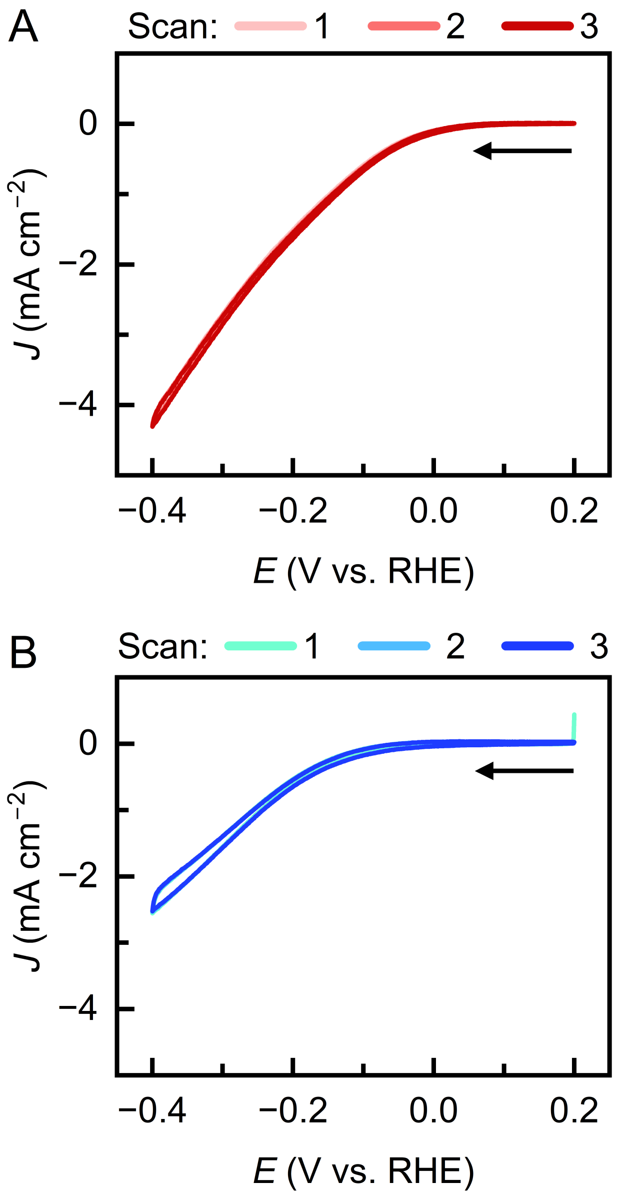

Figure S11. PFV scans of purified FDH on IO-TiO_2 electrodes (30 kDa filter). (A) Before and (B) after 10 h CPE at −0.4 V vs. RHE. Conditions: 50 mM $NaHCO_3$ + 50 mM KCl (CO_2-saturated, 25 ºC, pH 6.45), with stirring. FDH (500 pmol) was washed three times and re-concentrated with a 30 kDa filter prior to drop-casting on an IO-TiO_2 electrode. This was followed by co-immobilization with CA (100 pmol).

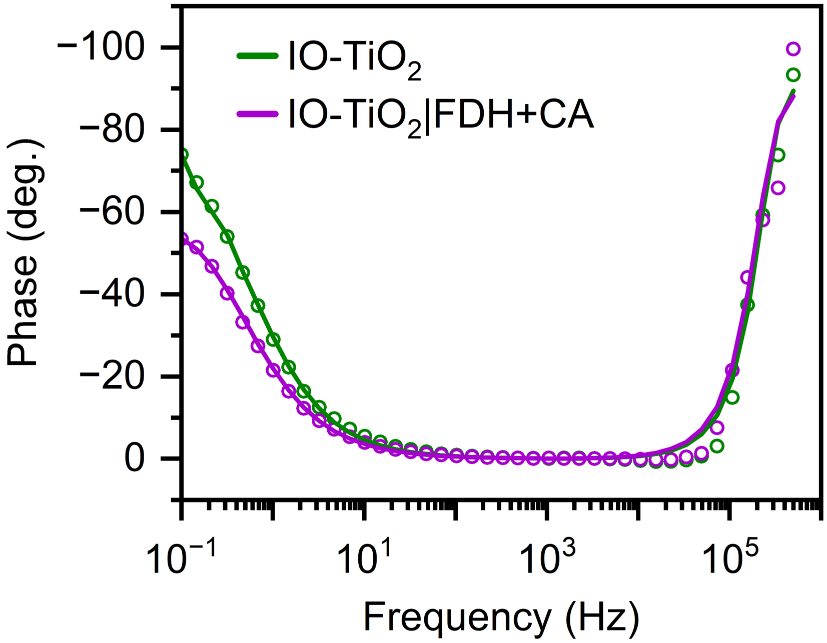

Figure S12. Electrochemical impedance analysis of an IO-TiO_2 electrode immobilized with purified FDH and CA. A bare IO-TiO_2 electrode is added for comparison. Conditions: 50 mM $NaHCO_3$ + 50 mM KCl (CO_2-saturated, 25 ºC, pH 6.45), with stirring.

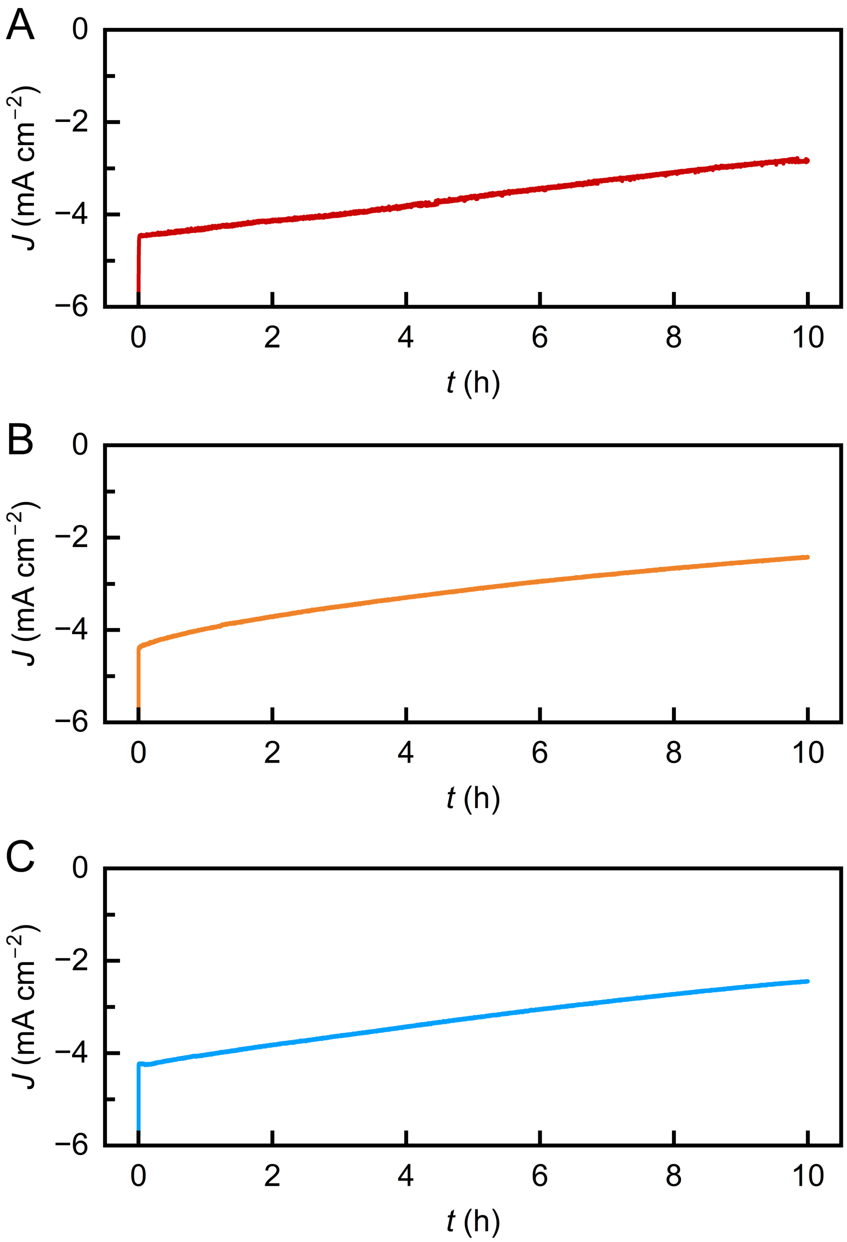

Figure S13. Reproducibility of IO-TiO_2|FDH+CA electrodes. (A-C) CPE traces recorded at −0.4 V vs. RHE over 10 h for 3 distinct samples (500 pmol purified FDH + 100 pmol CA). All experiments were performed in a 50 mM $NaHCO_3$ + 50 mM KCl buffer (CO_2-purged, pH 6.45, 25 ºC) with stirring.

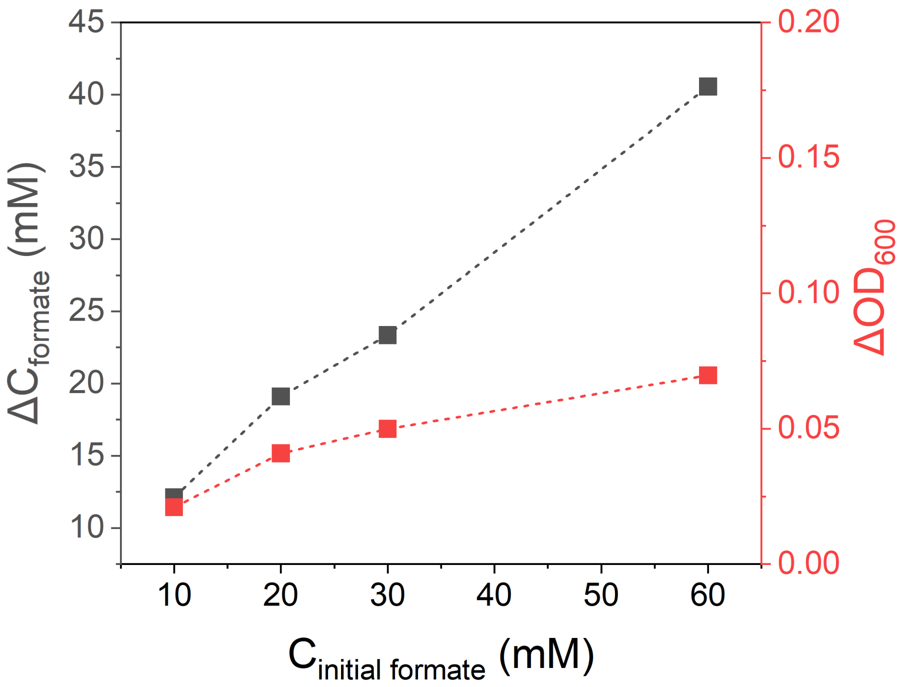

Figure S14. Initial formate concentration dictates autotrophic biomass yield. Biomass accumulation (ΔOD_{600}) of the strain cultured in preliminary batch experiments under initial formate concentrations ranging from 14 to 51 mM. While 51 mM formate yields the highest overall biomass accumulation, concentrations in the 20 to 30 mM range are sufficient to successfully support steady and measurable autotrophic growth, establishing the minimum requirements for the integrated system.

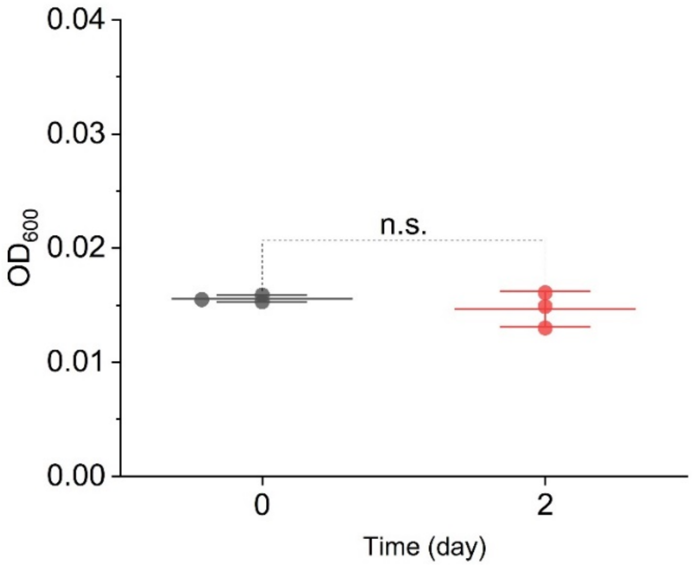

Figure S15. Trace element supplementation is essential for growth on electrochemically synthesized formate. Growth profile (OD_{600}) of the evolved *E. coli* strain cultivated in the electrolyte from the electrochemical system without the addition of supplementary trace elements. Under these conditions, no significant biomass accumulation was observed, demonstrating that trace element supplementation is required for autotrophic growth. Data are presented as the mean ± standard deviation from three biological replicates.

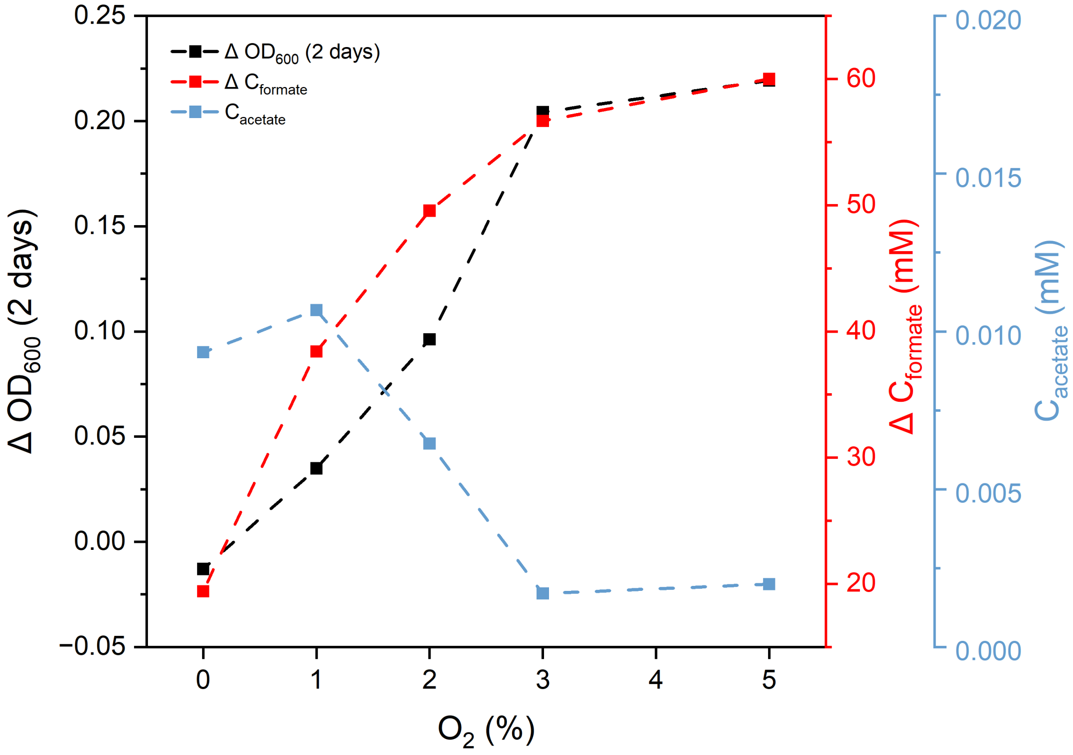

Figure S16. Oxygen concentration dictates autotrophic growth efficiency and alters fermentative metabolism. Growth profiles (OD_{600}), formate consumption, and acetate production of the evolved *E. coli* strain cultured under varying initial headspace oxygen concentrations. Decreasing oxygen levels led to a significant reduction in both the growth rate and final yield of biomass. At low oxygen concentrations (e.g., 1%), growth was severely inhibited and the culture exhibited a metabolic shift to fermentation, evidenced by the production and accumulation of acetate.

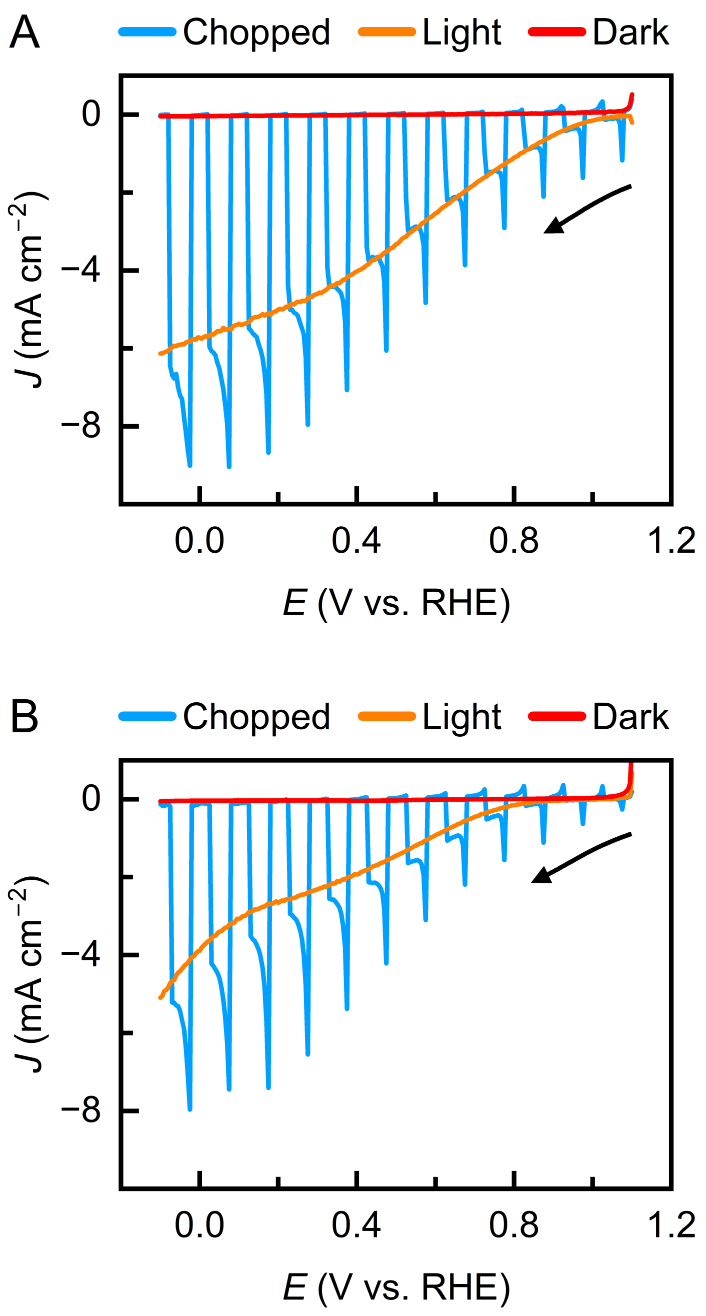

Figure S17. PFV scans of an OPV|IO-TiO_2|FDH+CA photocathode. (A) Before and (B) after 10 h CPE at 0.6 V vs. RHE. The traces were recorded at a scan rate of 10 mV s^{-1} under chopped, continuous and no illumination. Conditions: 1 sun irradiation (AM 1.5G, 100 mW cm^{-2}), 500 pmol purified FDH + 100 pmol CA, 50 mM $NaHCO_3$ + 50 mM KCl buffer (CO_2-purged, pH 6.45, 25 °C) with stirring.

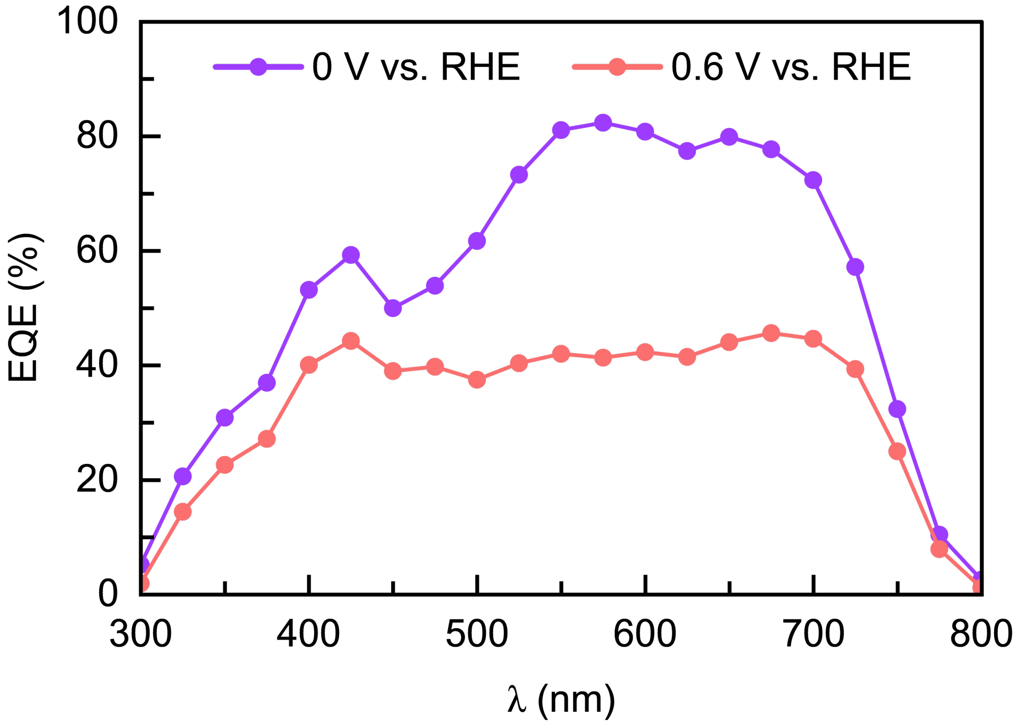

Figure S18. EQE spectra of the OPV|IO-TiO_2|FDH+CA photocathodes. EQE values are dependent on the applied potential, with 0 V vs. RHE corresponding to the standard condition (for direct comparison with other inorganic and organic photocathodes in the literature) and 0.6 V vs. RHE consistent with the applied potential on the photocathode during controlled potential electrolysis (**Figure S19**) and when paired with $BiVO_4$ in a bias-free configuration (**Figure S22**). Conditions: 50 mM $NaHCO_3$ + 50 mM KCl (CO_2-purged, 9 mL catholyte, with stirring, pH 6.45, room temperature), 500 pmol FDH + 100 pmol CA, simulated AM1.5G irradiation (100 mW cm^{-2}).

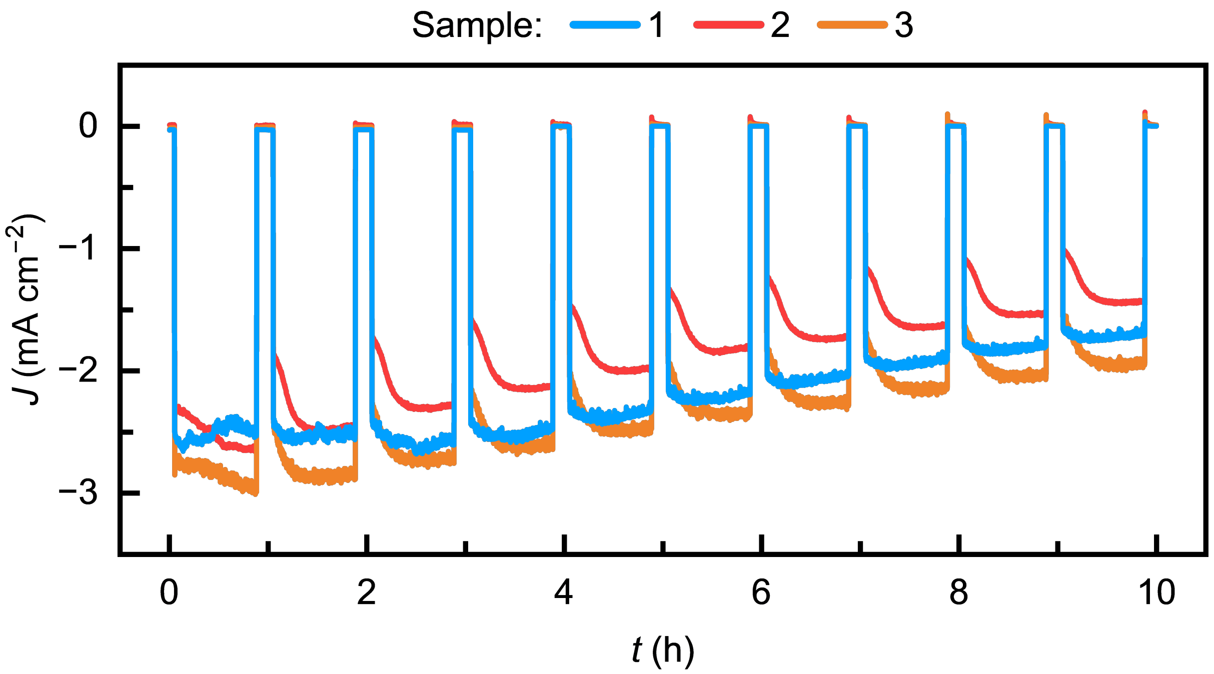

Figure S19. PEC performance of OPV|IO-TiO_2|FDH+CA photocathodes with purified FDH. Triplicate CPE scans recorded at 0.6 V vs. RHE for 10 h (50 min light on, 10 min light off cycles). Conditions: 500 pmol purified FDH + 100 pmol CA, 50 mM $NaHCO_3$ + 50 mM KCl buffer (CO_2-purged, pH 6.45, 25 ºC) with stirring, 1 sun irradiation (AM 1.5G, 100 mW cm^{-2}).

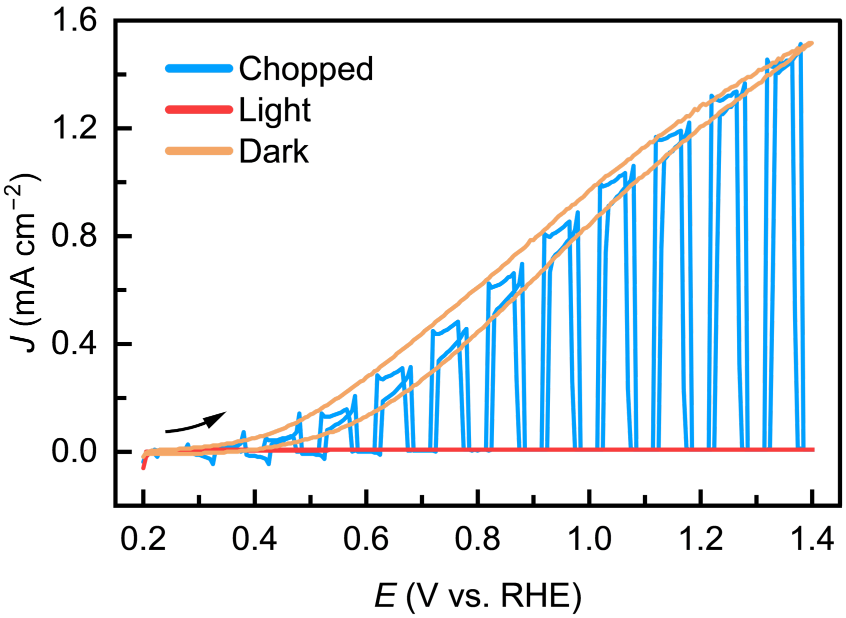

Figure S20. CV scans of the $BiVO_4$ photoanode. The traces were recorded under simulated 1 sun irradiation (AM1.5G, 100 mW cm^{-2}) in the presence of a UV filter (>400 nm) at 37 °C with continuous stirring. The electrolyte was 50 mM $NaHCO_3$ + 50 mM KCl (CO_2-purged, pH 6.45).

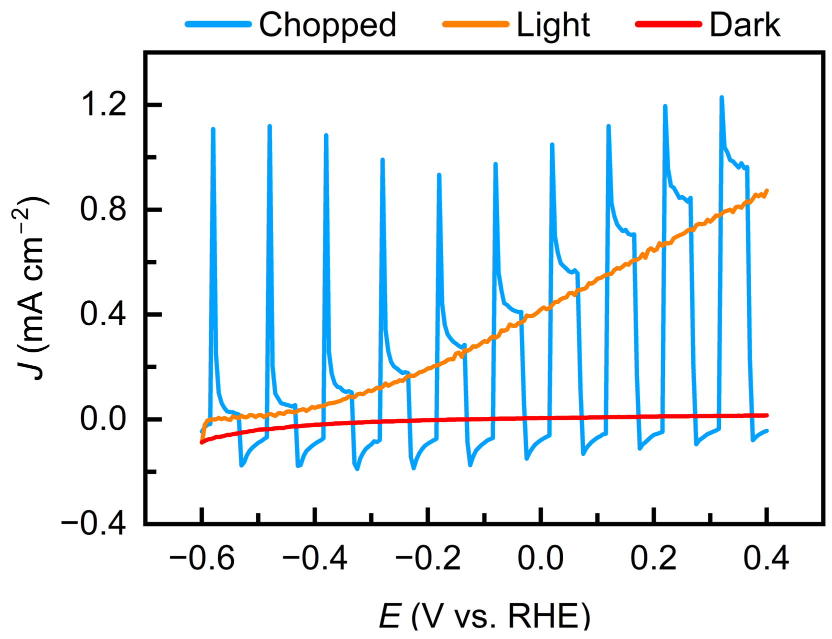

Figure S21. PFV scans for a semi-artificial photoelectrochemical leaf integrated with self-sustaining autotrophic cultivation. The traces were recorded at a scan rate of 10 mV s^{-1} under chopped, continuous and dark illumination in a two-electrode system. An early onset voltage of −0.6 V was observed. Conditions: 50 mM $NaHCO_3$ + 50 mM KCl electrolyte (CO_2-purged, pH 6.45) containing *E. coli* at 37 ºC with continuous stirring, AM1.5G irradiation (100 mW cm^{-2}) with UV filter.

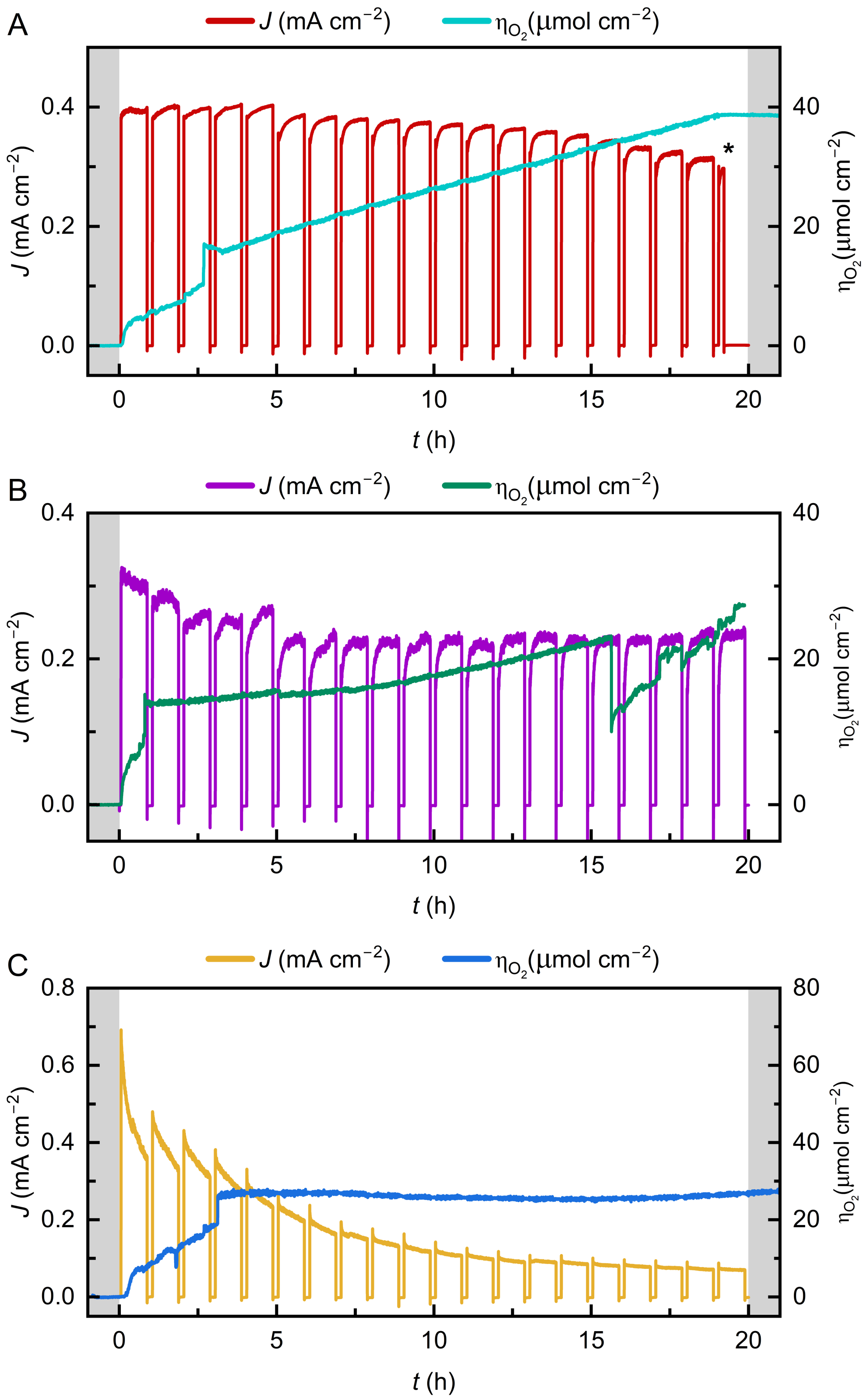

Figure S22. PEC triplicates for the semi-artificial leaf. (A-C) CPE and O_2 evolution traces recorded under bias-free conditions for 3 distinct samples (500 pmol purified FDH and 100 pmol CA). All experiments were performed under 1 sun irradiation (AM 1.5G, 100 mW cm^{-2}) in a CO_2-purged 50 mM $NaHCO_3$ + 50 mM KCl buffer (pH 6.45) containing *E. coli* at 37 °C with continuous stirring. The decrease in photocurrent density at 19 h as shown in (A) was due to an electrical connection issue. The noisy O_2 signal in (B) could be attributed to water condensation on the tip of the fluorescence sensor, further accelerated by heating the reaction at elevated temperatures.[1]

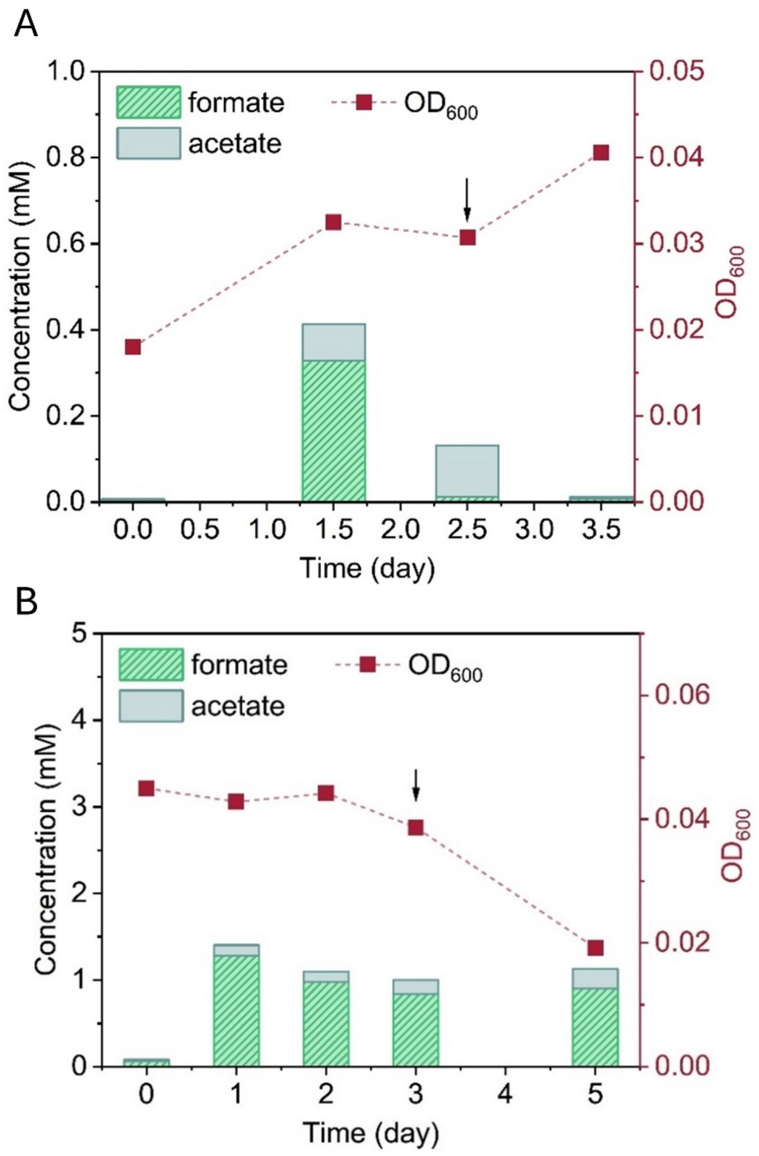

Figure S23. Trace elements are essential for microbial growth but inhibit initial PEC formate production. Comparison of microbial growth (OD_{600}) and formate concentration in the integrated semi-artificial leaf system under two conditions. (A) In the presence of supplementary trace elements and (B) in the absence of trace elements from the start. Arrows indicate the exchange of headspace gas from 100% CO_2 into 5% O_2, 10% CO_2, and balanced N_2.

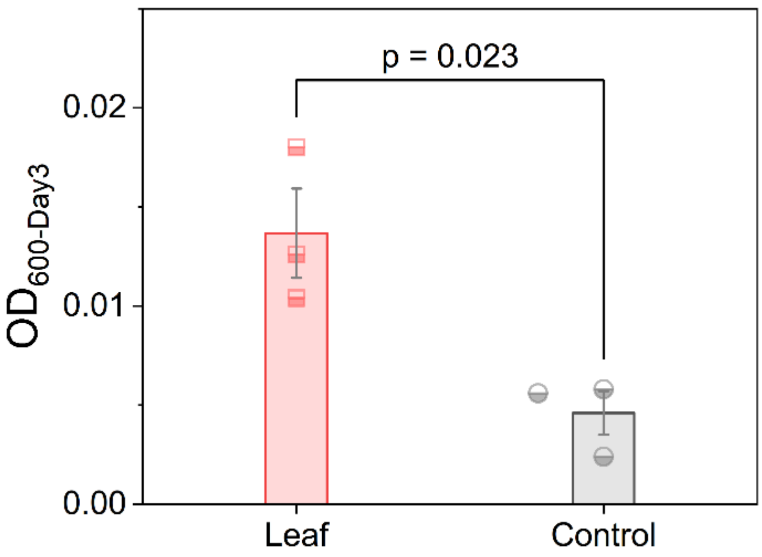

Figure S24. Statistical analysis of light-dependent biomass production. Final biomass, measured as optical density (OD_{600}), of *E. coli* cultures in the integrated semi-artificial leaf system after three days. The bar chart compares the final cell density of the system operated under simulated solar illumination ('Leaf') versus an identical system kept in constant darkness ('Control'). The light-driven culture shows a statistically significant increase in biomass compared to the dark control ($p = 0.023$). Error bars represent the standard deviation from three biological replicates.

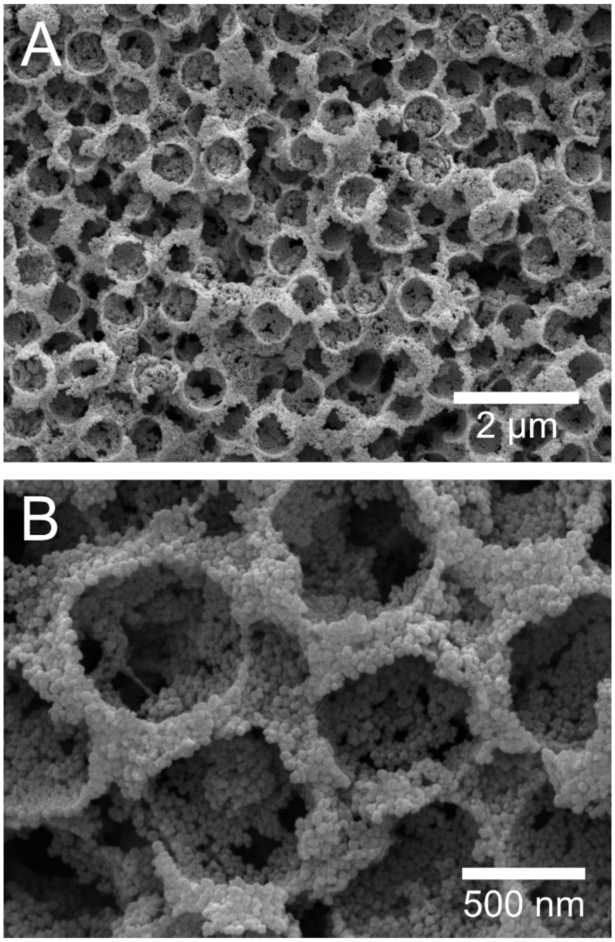

Figure S25. Scanning electron microscopy (SEM) images of the IO-TiO_2 electrode in a semi-artificial leaf device after biomass production. The images were recorded at view fields of (A) 10 µm and (B) 2.5 µm using a 5 kV electron beam accelerating voltage. IO-TiO_2 retained its hierarchically porous morphology, and no biofilm formation was observed after the three-day experiment.

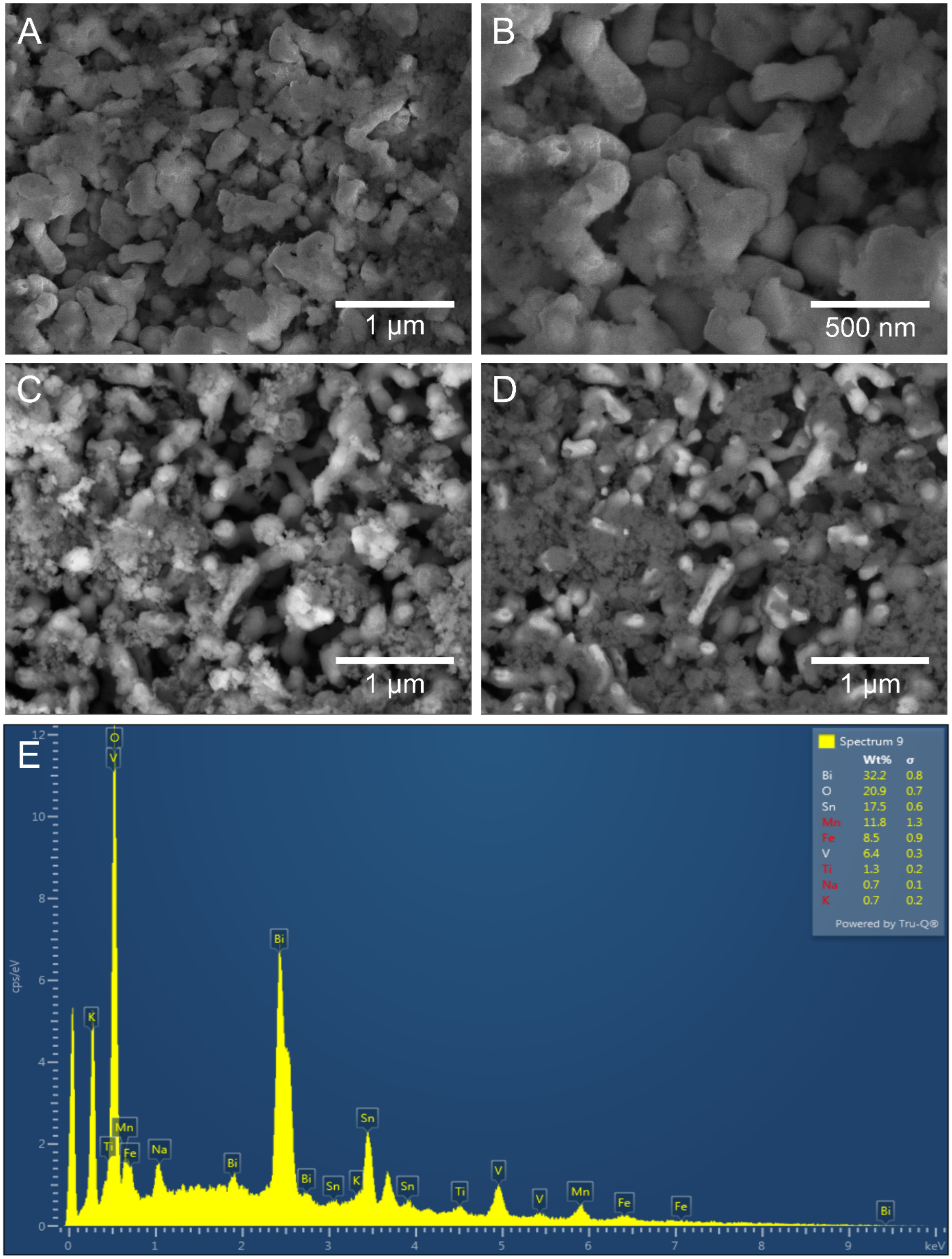

Figure S26. SEM images of the $BiVO_4$|TiCo photoanode from the tandem device post-incubation with *E. coli* for 3 days. (A-B) SEM images recorded at view fields of (A) 4 µm and (B) 2 µm. (C-D) Further analysis using backscattered electron (BSE) imaging for better image contrast with (C) secondary electrons and (D) back-scattered electrons. Brighter regions in the BSE images indicate areas of elements with heavier atomic masses. (E) Energy-dispersive X-ray (EDX) spectrum. Salt deposits are presumably observed from the bacteria media on the surface of the $BiVO_4$ photoanode.

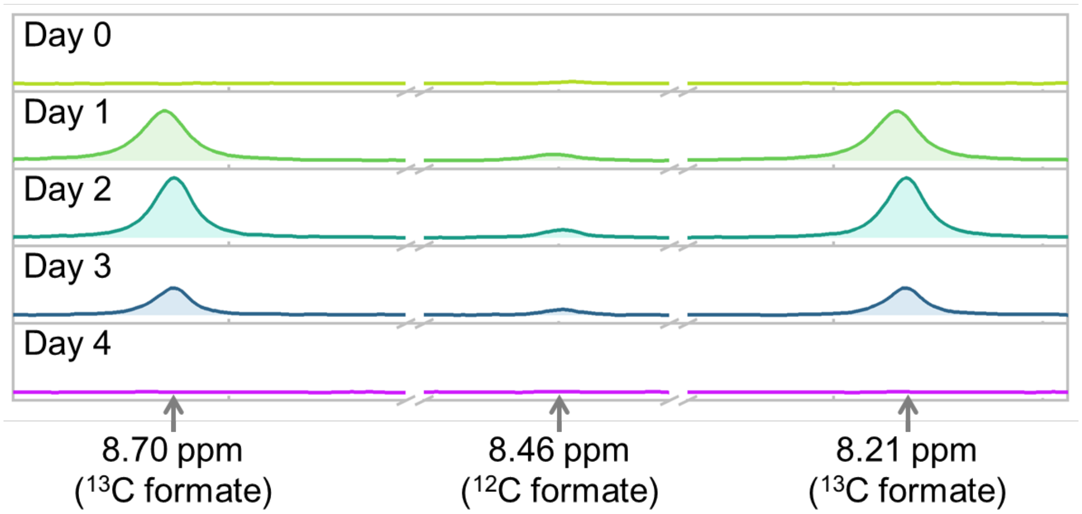

Figure S27. NMR spectroscopic analysis of isotopic labelling. ^{1}H NMR spectra of the electrolyte from the integrated semi-artificial leaf experiment, which was supplied with $^{13}CO_2$ as the carbon feedstock. The spectra show the time-dependent emergence and subsequent consumption of the characteristic doublet signal for ^{13}C-formate, which is distinct from the singlet for naturally abundant ^{12}C-formate. This result confirms that the formate produced by the device originates from the supplied $^{13}CO_2$ and is then consumed by the microbial culture.

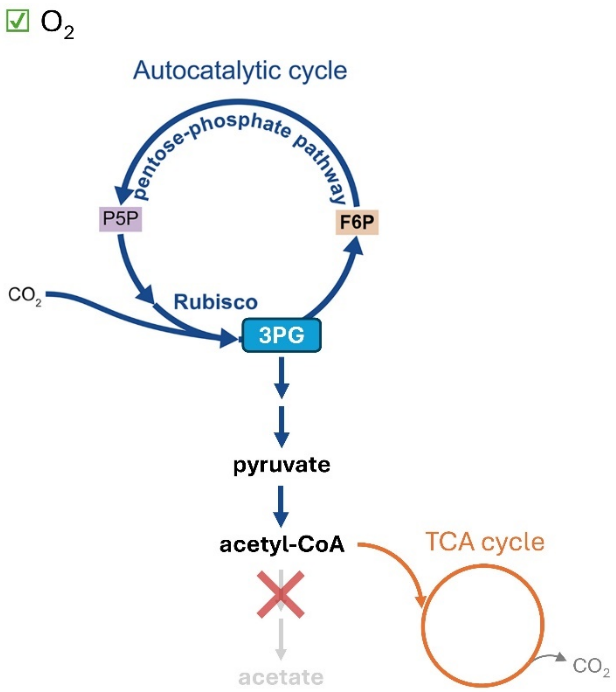

Figure S28. Proposed metabolic pathway for acetate synthesis under hypoxic conditions.

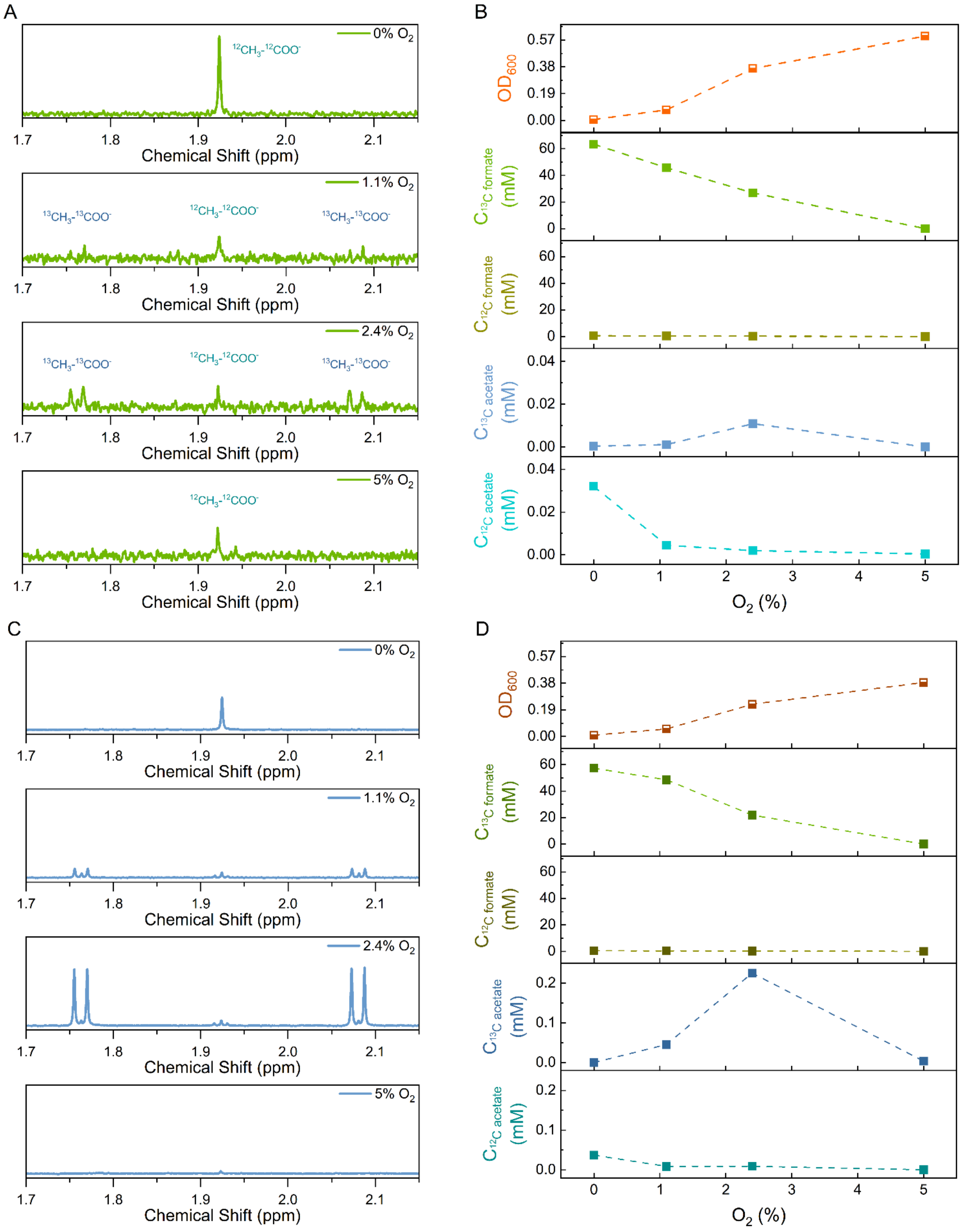

Figure S29. Isotope tracing confirms acetate is synthesized from fixed carbon under hypoxic conditions. Representative ^{1}H NMR spectra of the bacterial growth medium after (A) two days and (C) thirteen days incubation. Cultures were supplied with ^{13}C-labelled HCO_3^- and ^{13}C-labelled formate as sole carbon sources under headspace atmospheres containing 0–5% O_2 (balanced with N_2). Quantification of final biomass (OD_{600}) and the amounts of $^{12}C/^{13}C$-formate and $^{12}C/^{13}C$-acetate at the end of the (B) two days and (D) thirteen days incubation, plotted as a function of the initial headspace O_2 concentration.

Table S1. Bacterial utilization of formate for CO_2 assimilation.

Host organism	*Pathway / module introduced*	*Energy mode*	*Coupling topology*	*Ref.*
Escherichia coli (engineered)	Heterologous Calvin–Benson–Bassham (CBB) module (Rubisco + phosphoribulokinase) combined with a heterologous formate dehydrogenase	Formate oxidation	Added external formate	17–19
Escherichia coli (engineered)	Synthetic reductive glycine pathway (rGlyP)	Formate oxidation	Added external formate	20,21
Escherichia coli (engineered)	Fast, metal-dependent formate dehydrogenase complex with rGlyP	Formate oxidation	Electrochemically generated formate, disintegrate system	22
Escherichia coli (engineered)	Tetrahydrofolate and serine cycles	Formate assimilation	external formate and glycine	23
Escherichia coli (engineered)	Solar formic acid/pentose (SFAP) pathway	Solar and glucose oxidation	Artificial PEC compartment for formate synthesis, integrate with separate bio-chamber	24
Escherichia coli (engineered)	Synthetic Serine Threonine Cycle (STC)	Formate oxidation	Added external formate	25
Escherichia coli (engineered)	Formyl-CoA elongation (FORCE) reactions	Formate assimilation	Added external formate	26
Escherichia coli (engineered)	Reconstructed tetrahydrofolate (THF) cycle, reverse glycine cleavage reaction and *Candida boidinii* Fdh (CbFdh)	Formate oxidation	Added external formate	27
Cupriavidus necator (engineered)	Replace native Calvin cycle with the reductive glycine pathway	Formate oxidation	Added external formate	28
Cupriavidus necator	Native Wood–Ljungdahl pathway (WLP)	Formate oxidation	Electrochemical CO_2 reduction, integrate with separate bio-chamber	29
Clostridium ljungdahlii	Native Wood–Ljungdahl pathway (WLP)	Formate and syngas (CO, H_2, CO_2)	Disintegrate with external formate; integrated with syngas photosynthesis with separate bio-chamber	11

Table S2. Whole genome sequencing results after adaptive lab evolution

Ancestor_Transfer_#1						
**evidence**	**seq id**	**position**	**mutation**	**annotation**	**gene**	**description**
RA	CP009273	33,395	C→A	P860Q (CCG→CAG)	*carB* →	carbamoyl-phosphate synthase large subunit
RA	CP009273	3,479,542	C→A	H22N (CAT→AAT)	*crp* →	cAMP-activated global transcription factor, mediator of catabolite repression
MC JC	CP009273	2400028	Δ9 bp	coding (85-93/939 nt)	*lrhA* ←	transcriptional repressor of flagellar, motility and chemotaxis genes
RA	CP009273	4205290	A→C	intergenic (+153/-116)	*metA* → / → *aceB*	homoserine O-transsuccinylase/malate synthase A
RA	CP009273	4224841	C→T	H386Y (CAC→TAC)	*pgi* →	glucosephosphate isomerase
RA	CP009273	4174906	C→T	A1245V (GCG→GTG)	*rpoB* →	RNA polymerase, beta subunit
RA	CP009273	3466971	A→T	L157* (TTA→TAA)	*rpsG* ←	30S ribosomal subunit protein S7
JC	CP009273	2791731	(TTC)4→5	coding (124/405 nt)	*stpA* ←	DNA binding protein, nucleoid-associated
JC	pZS2M_pseudoFDH	918	Δ8 bp	intergenic (-126/-86)	*strepR* ← / → *pseudFDH*	strepR/pseudFDH
RA	CP009273	3840509	G→A	Q7* (CAG→TAG)	*uhpT* ←	hexose phosphate transporter
JC	CP009273	2271667	(AGACCAGC)1→2	coding (50/345 nt)	*yejG* ←	uncharacterized protein

Evolved_Transfer_#27						
**evidence**	**seq id**	**position**	**mutation**	**annotation**	**gene**	**description**
RA	CP009273	33,395	C→A	P860Q (CCG→CAG)	*carB* →	carbamoyl-phosphate synthase large subunit
RA	CP009273	3,479,542	C→A	H22N (CAT→AAT)	*crp* →	cAMP-activated global transcription factor, mediator of catabolite repression
MC JC	CP009273	2,400,028	Δ9 bp	coding (85-93/939 nt)	*lrhA* ←	transcriptional repressor of flagellar, motility and chemotaxis genes
RA	CP009273	4,205,290	A→C	intergenic (+153/-116)	*metA* → / → *aceB*	homoserine O-transsuccinylase/malate synthase A
RA	CP009273	4,224,841	C→T	H386Y (CAC→TAC)	*pgi* →	glucosephosphate isomerase
RA	CP009273	3,632,458	"+T"	coding (1457/1500 nt)	*pitA* →	phosphate transporter, low-affinity; tellurite importer
RA	CP009273	4,174,906	C→T	A1245V (GCG→GTG)	*rpoB* →	RNA polymerase, beta subunit
RA	CP009273	3,466,971	A→T	L157* (TTA→TAA)	*rpsG* ←	30S ribosomal subunit protein S7
JC	CP009273	2,791,731	(TTC)4→5	coding (124/405 nt)	*stpA* ←	DNA binding protein, nucleoid-associated
JC	pZS2M_pseudoFDH	918	Δ8 bp	intergenic (-126/-86)	*strepR* ← / → *pseudFDH*	strepR/pseudFDH
RA	CP009273	3,840,509	G→A	Q7* (CAG→TAG)	*uhpT* ←	hexose phosphate transporter
JC	CP009273	2,271,667	(AGACCAGC)1→2	coding (50/345 nt)	*yejG* ←	uncharacterized protein

Table S3. Oxygen production during semi-artificial leaf experiments.

	Headspace (V = 9.7 mL)		Electrolyte (V = 5mL)
	O_2 (%)	η_{O2} (μmol)	η_{O2} (μmol)
Replicate 1	0.8	3.2	0.1
Replicate 2	2.5	9.9	0.2
Replicate 3	1.5	6.1	0.1

Table S4. Comparison with state-of-the-art bio-photoelectrochemical devices

Device structure	Reaction	E_{onset}/ V	J/ mA cm^{-2}	Stability	FE/ %	STF/ %	Conditions	Ref.
**Photocathode only**								
Ni foam|NiO|PAA@NHS|Au|*A. succinogenes*[a]	CO_2R-succinic acid	0	–1.9 at −0.3 V_{RHE}	12 h (~100%) at −0.3 V_{RHE}	57	-	Fermentation broth (pH 7)	30
FTO|$CuBi_2O_4$|MgO|*S. ovata* → *C. kluyveri*	CO_2R-C_2-C_4 products	1.0	–0.07 at 0.4 V_{RHE}	140 h (~145%) at 0.4 V_{RHE}	69	-	*S. ovata* medium (pH 7)	31
n^+p-Si NWs|TiO_2|Pt|*S. ovata*[c]	CO_2R-acetate	0.4	–0.75 at 0.2 V_{RHE}[d]	60 h (~500%) at 0.2 V_{RHE}	80	-	Bacterial medium + 50 mM MES (pH 6.2)	32
ITO|PEDOT:PSS|PCE10:EH-IDTBR|ZnO|Ag|GE|Ti foil|IO-TiO_2|FDH+CA → *E. coli*	CO_2R-formate-biomass	1.0	–3 at 0.6 V_{RHE}	10 h (66%) at 0.6 V_{RHE}	97	-	50 mM $NaHCO_3$, 50 mM KCl (pH 6.45)	This work
**Photovoltaic-Electrolyzer (PV-EC)**								
Ti foil|*p*-Si NWs|TiO_2|Ni|*S. ovata*[e] || Pt wire → *R. palustris nifA**	CO_2R-acetate-PHB||OER[f]	-	-	48 h (–)[g]	-	1.78	Photoheterotrophic medium (pH 6.8)	33
**Dual light-absorber PEC tandems**								
p-Si NWs|TiO_2|Ni|*S. ovata* || FTO|TiO_2 → *E. coli*	CO_2R-acetate-PHB||OER	-	0.3	120 h (71%) at 0 V	86	0.38	M9-MOPS[h] (pH 7)	34
n^+p-Si NWs|TiO_2|Pt|*S. ovata* || *n*-Si NWs|Pt-Au	CO_2R-acetate||GOR[i]	–0.8	1.2[j]	5 h (62%) at 0 V	87	-	Cathode: Bacterial medium + 50 mM MES (pH 6.2) Anode: 1 M KOH	32
PTB7-Th:EH-IDTBR OPV|GE|Ti foil|IO-TiO_2|FDH+CA || FTO|$BiVO_4$|TiCo → *E. coli*	CO_2R-formate-biomass||OER	–0.6	0.4	20 h (76%) at 0 V	97	0.45	50 mM $NaHCO_3$, 50 mM KCl (pH 6.45)	This work

The relevant performance metrics are: E_{onset} (onset potential vs. RHE for photoelectrodes or absolute onset potential for tandem devices), J (steady state or average photocurrent measured at an applied potential for photoelectrodes or under bias-free conditions for tandem devices), stability (duration of controlled potential electrolysis and percentage of initial photocurrent density remaining afterwards), FE (Faradaic efficiency for the chemical intermediate and not the final product, unless there is only one product) and STF (solar-to-fuel conversion efficiency for the chemical intermediate). Chemical intermediates (e.g. C_2 products, formate, acetate) were chosen as the basis for comparison, considering the different microbial metabolic rates across various strains for the final product. All experiments were performed under 1 sun illumination (AM 1.5G, 100 mW cm^{-2}) unless stated otherwise. For the device architecture, microbes/enzymes immobilized directly on the photoelectrode are denoted by a vertical bar symbol (|), while those suspended in solution regardless of a one-pot or a two-step consecutive reaction are denoted an arrow symbol (→). [a] Photoelectrode consisting of NiO nanosheets coated with a poly acrylic acid (PAA) hydrogel grafted with N-hydroxysuccinimide (NHS) groups. [c] A n^+ shell was coated onto the *p*-type Si NW array to increase photovoltage. [d] Low-intensity red light (740 nm) at 20 mW cm^{-2} was used. [e] PV-EC configuration where a commercially available multijunction Si solar cell was connected (open-circuit voltage = 4.7 V, short-circuit photocurrent = 4.4 mA under 1 sun illumination). [f] PHB: polyhydroxybutyrate. OER: oxygen evolution reaction. [g] Light intensity on the solar cell was set at 25 mW cm^{-2}. [h] M9 minimal medium is a buffer commonly used for bacteria cultivation, containing many salts and minerals such as phosphates, sulfates and vitamins. MOPS: 3-(N-morpholino)propanesulfonic acid. [i] GOR: glycerol oxidation reaction. [j] Low-intensity red light (740 nm) at 20 mW cm^{-2} was used.

Supporting References

(1) Grenier, F.; Matteau, D.; Baby, V.; Rodrigue, S. Complete Genome Sequence of Escherichia Coli BW25113. *Genome Announc.* **2014**, *2* (5), e01038-14. https://doi.org/10.1128/genomea.01038-14.

(2) Deatherage, D. E.; Barrick, J. E. Identification of Mutations in Laboratory-Evolved Microbes from next-Generation Sequencing Data Using Breseq. *Methods Mol. Biol. (Clifton, NJ)* **2014**, *1151*, 165–188. https://doi.org/10.1007/978-1-4939-0554-6_12.

(3) Miller, J. H. *A Short Course in Bacterial Genetics*; Cold Spring Harbor Laboratory Press, 1992.

(4) Baba, T.; Ara, T.; Hasegawa, M.; Takai, Y.; Okumura, Y.; Baba, M.; Datsenko, K. A.; Tomita, M.; Wanner, B. L.; Mori, H. Construction of Escherichia Coli K-12 In-frame, Single-gene Knockout Mutants: The Keio Collection. *Mol. Syst. Biol.* **2006**, *2* (1), MSB4100050. https://doi.org/10.1038/msb4100050.

(5) Gallagher, R. R.; Li, Z.; Lewis, A. O.; Isaacs, F. J. Rapid Editing and Evolution of Bacterial Genomes Using Libraries of Synthetic DNA. *Nat. Protoc.* **2014**, *9* (10), 2301–2316. https://doi.org/10.1038/nprot.2014.082.

(6) Yeung, C. W. S.; Liu, Y.; Cobb, S.; Andrei, V.; Coito, A.; Manuel, R.; Pereira, I.; Reisner, E. Semi-Artificial Leaf Interfacing Organic Semiconductors and Enzymes for Solar Fuel Synthesis. **2024**. https://doi.org/10.26434/chemrxiv-2024-f49zl.

(7) Moore, E. E.; Andrei, V.; Oliveira, A. R.; Coito, A. M.; Pereira, I. A. C.; Reisner, E. A Semi-artificial Photoelectrochemical Tandem Leaf with a CO_2-to-Formate Efficiency Approaching 1%. *Angew. Chem. Int. Ed.* **2021**, *60* (50), 26303–26307. https://doi.org/10.1002/anie.202110867.

(8) Yeung, C. W. S.; Andrei, V.; Lee, T. H.; Durrant, J. R.; Reisner, E. Organic Semiconductor-$BiVO_4$ Tandem Devices for Solar-Driven H_2O and CO_2 Splitting. *Adv. Mater.* **2024**, *36* (35), e2404110. https://doi.org/10.1002/adma.202404110.

(9) Andrei, V.; Hoye, R. L. Z.; Crespo-Quesada, M.; Bajada, M.; Ahmad, S.; Volder, M. D.; Friend, R.; Reisner, E. Scalable Triple Cation Mixed Halide Perovskite–$BiVO_4$ Tandems for Bias-Free Water Splitting. *Adv. Energy Mater.* **2018**, *8* (25), 1801403. https://doi.org/10.1002/aenm.201801403.

(10) Lai, Y.; Palm, D. W.; Reisner, E. Multifunctional Coatings from Scalable Single Source Precursor Chemistry in Tandem Photoelectrochemical Water Splitting. *Adv. Energy Mater.* **2015**, *5* (24). https://doi.org/10.1002/aenm.201501668.

(11) Su, L.; Rodríguez-Jiménez, S.; Short, M. I. M.; Reisner, E. Adapting Gas Fermenting Bacteria for Light-Driven Domino Valorization of CO_2. *Chem. Sci.* **2025**, *16* (26), 11801–11808. https://doi.org/10.1039/d5sc00764j.

(12) Bouwens, T.; Cobb, S. J.; Yeung, C. W. S.; Liu, Y.; Martins, G.; Pereira, I. A. C.; Reisner, E. Semiartificial Photoelectrochemistry for CO_2 Mediated Enantioselective Organic Synthesis. *J. Am. Chem. Soc.* **2025**, *147* (16), 13114–13119. https://doi.org/10.1021/jacs.5c02250.

(13) Low, B. Q. L.; Rodríguez-Jiménez, S.; Rogolino, A.; Cobb, S.; Han, C.; Martins, G.; Pereira, I.; Reisner, E. Enzymatic Flow Electrolyser for the Comproportionation of CO_2 and Organic Waste to Formate. **2025**. https://doi.org/10.26434/chemrxiv-2025-8hc25.

(14) Oliveira, A. R.; Mota, C.; Mourato, C.; Domingos, R. M.; Santos, M. F. A.; Gesto, D.; Guigliarelli, B.; Santos-Silva, T.; Romão, M. J.; Pereira, I. A. C. Toward the Mechanistic Understanding of Enzymatic CO_2 Reduction. *ACS Catal.* **2020**, *10* (6), 3844–3856. https://doi.org/10.1021/acscatal.0c00086.

(15) Murray, K. A.; Gibson, M. I. Chemical Approaches to Cryopreservation. *Nat. Rev. Chem.* **2022**, *6* (8), 579–593. https://doi.org/10.1038/s41570-022-00407-4.

(16) Yeung, C. W. S.; Liu, Y.; Vahey, D. M.; Cobb, S. J.; Andrei, V.; Coito, A. M.; Manuel, R. R.; Pereira, I. A. C.; Reisner, E. Semi-Artificial Leaf Interfacing Organic Semiconductors and Enzymes for Solar Chemical Synthesis. *Joule* **2025**, *9* (11), 102165. https://doi.org/10.1016/j.joule.2025.102165.

(17) Antonovsky, N.; Gleizer, S.; Noor, E.; Zohar, Y.; Herz, E.; Barenholz, U.; Zelcbuch, L.; Amram, S.; Wides, A.; Tepper, N.; Davidi, D.; Bar-On, Y.; Bareia, T.; Wernick, D. G.; Shani, I.; Malitsky, S.; Jona, G.; Bar-Even, A.; Milo, R. Sugar Synthesis from CO_2 in Escherichia Coli. *Cell* **2016**, *166* (1), 115–125. https://doi.org/10.1016/j.cell.2016.05.064.

(18) Gleizer, S.; Ben-Nissan, R.; Bar-On, Y. M.; Antonovsky, N.; Noor, E.; Zohar, Y.; Jona, G.; Krieger, E.; Shamshoum, M.; Bar-Even, A.; Milo, R. Conversion of Escherichia Coli to Generate All Biomass Carbon from CO_2. *Cell* **2019**, *179* (6), 1255-1263.e12. https://doi.org/10.1016/j.cell.2019.11.009.

(19) Nissan, R. B.; Milshtein, E.; Pahl, V.; Pins, B. de; Jona, G.; Levi, D.; Yung, H.; Nir, N.; Ezra, D.; Gleizer, S.; Link, H.; Noor, E.; Milo, R. Autotrophic Growth of Escherichia Coli Is Achieved by a Small Number of Genetic Changes. *eLife* **2024**, *12*, RP88793. https://doi.org/10.7554/elife.88793.

(20) Kim, S.; Lindner, S. N.; Aslan, S.; Yishai, O.; Wenk, S.; Schann, K.; Bar-Even, A. Growth of E. Coli on Formate and Methanol via the Reductive Glycine Pathway. *Nat. Chem. Biol.* **2020**, *16* (5), 538–545. https://doi.org/10.1038/s41589-020-0473-5.

(21) Delmas, V. A.; Perchat, N.; Monet, O.; Fouré, M.; Darii, E.; Roche, D.; Dubois, I.; Pateau, E.; Perret, A.; Döring, V.; Bouzon, M. Genetic and Biocatalytic Basis of Formate Dependent Growth of Escherichia Coli Strains Evolved in Continuous Culture. *Metab. Eng.* **2022**, *72*, 200–214. https://doi.org/10.1016/j.ymben.2022.03.010.

(22) Cowan, A. E.; Hillers, M.; Rainaldi, V.; Collas, F.; Choudhary, H.; Zakaria, B. S.; Bieberach, G. G.; Carruthers, D. N.; Grabovac, M.; Gin, J. W.; Cawthon, B.; Chen, Y.; Turumtay, E. A.; Baidoo, E. E. K.; Petzold, C. J.; Feist, A. M.; Tejedor-Sanz, S.; Kensy, F.; Simmons, B. A.; Keasling, J. D.; Claassens, N. J. Fast Growth and High-Titer Bioproduction from Renewable Formate via Metal-Dependent Formate Dehydrogenase in Escherichia Coli. *Nat. Commun.* **2025**, *16* (1), 5908. https://doi.org/10.1038/s41467-025-61001-y.

(23) Kim, S.-J.; Yoon, J.; Im, D.-K.; Kim, Y. H.; Oh, M.-K. Adaptively Evolved Escherichia Coli for Improved Ability of Formate Utilization as a Carbon Source in Sugar-Free Conditions. *Biotechnol. Biofuels* **2019**, *12* (1), 207. https://doi.org/10.1186/s13068-019-1547-z.

(24) Zhang, Y.; Sun, T.; Liu, L.; Cao, X.; Zhang, W.; Wang, W.; Li, C. Engineering a Solar Formic Acid/Pentose (SFAP) Pathway in Escherichia Coli for Lactic Acid Production. *Metab. Eng.* **2024**, *83*, 150–159. https://doi.org/10.1016/j.ymben.2024.04.002.

(25) Wenk, S.; Rainaldi, V.; Schann, K.; He, H.; Bouzon, M.; Döring, V.; Lindner, S. N.; Bar-Even, A. Evolution-Assisted Engineering of E. Coli Enables Growth on Formic Acid at Ambient CO_2 via the Serine Threonine Cycle. *Metab. Eng.* **2025**, *88*, 14–24. https://doi.org/10.1016/j.ymben.2024.10.007.

(26) Chou, A.; Lee, S. H.; Zhu, F.; Clomburg, J. M.; Gonzalez, R. An Orthogonal Metabolic Framework for One-Carbon Utilization. *Nat. Metab.* **2021**, *3* (10), 1385–1399. https://doi.org/10.1038/s42255-021-00453-0.

(27) Bang, J.; Hwang, C. H.; Ahn, J. H.; Lee, J. A.; Lee, S. Y. Escherichia Coli Is Engineered to Grow on CO_2 and Formic Acid. *Nat. Microbiol.* **2020**, *5* (12), 1459–1463. https://doi.org/10.1038/s41564-020-00793-9.

(28) Dronsella, B.; Orsi, E.; Schulz-Mirbach, H.; Benito-Vaquerizo, S.; Yilmaz, S.; Glatter, T.; Bar-Even, A.; Erb, T. J.; Claassens, N. J. One-Carbon Fixation via the Synthetic Reductive Glycine Pathway Exceeds Yield of the Calvin Cycle. *Nat. Microbiol.* **2025**, *10* (3), 646–653. https://doi.org/10.1038/s41564-025-01941-9.

(29) Lim, J.; Choi, S. Y.; Lee, J. W.; Lee, S. Y.; Lee, H. Biohybrid CO_2 Electrolysis for the Direct Synthesis of Polyesters from CO_2. *Proc. Natl. Acad. Sci.* **2023**, *120* (14), e2221438120. https://doi.org/10.1073/pnas.2221438120.

(30) Feng, T.; Zhou, X.; Zhang, Y.; Zhang, Z. Photoelectrocatalytic-Microbial Biohybrid for Succinic Acid Synthesis. *Nat. Commun.* **2026**, *17* (1), 3112. https://doi.org/10.1038/s41467-026-69962-4.

(31) Burns, C.; Rishan, M.; Stevens, L.; Ashcroft, E.; Fuller, L.; Gibson, E. A.; Kalathil, S. A Living Semiartificial Photoelectrocatalytic Biohybrid for Solar CO_2 Fixation and Fermentation to Fatty Acids. *ACS Appl. Mater. Interfaces* **2025**, *17* (46), 63363–63373. https://doi.org/10.1021/acsami.5c15023.

(32) Kim, J.; Lin, J.-A.; Kim, J.; Roh, I.; Lee, S.; Yang, P. A Red-Light-Powered Silicon Nanowire Biophotochemical Diode for Simultaneous CO_2 Reduction and Glycerol Valorization. *Nat. Catal.* **2024**, *7* (9), 977–986. https://doi.org/10.1038/s41929-024-01198-1.

(33) Cestellos-Blanco, S.; Chan, R. R.; Shen, Y.; Kim, J. M.; Tacken, T. A.; Ledbetter, R.; Yu, S.; Seefeldt, L. C.; Yang, P. Photosynthetic Biohybrid Coculture for Tandem and Tunable CO_2 and N_2 Fixation. *Proc. Natl. Acad. Sci.* **2022**, *119* (26), e2122364119. https://doi.org/10.1073/pnas.2122364119.

(34) Liu, C.; Gallagher, J. J.; Sakimoto, K. K.; Nichols, E. M.; Chang, C. J.; Chang, M. C. Y.; Yang, P. Nanowire–Bacteria Hybrids for Unassisted Solar Carbon Dioxide Fixation to Value-Added Chemicals. *Nano Lett.* **2015**, *15* (5), 3634–3639. https://doi.org/10.1021/acs.nanolett.5b01254.

End of Supporting Information
